# Astroglial disinhibition of cortical circuits disrupts cognition via kynurenic acid in mice

**DOI:** 10.1038/s41467-026-72640-0

**Published:** 2026-05-08

**Authors:** Viktor Beilmann, Johanna Furrer, Sina M. Schalbetter, Ron Schaer, Edoardo Tiziani, Kim D. Ferrari, Felisa Herrero, Celine Heeb, Alexandra von Faber-Castell, Jacqueline Condrau, Ulrike Weber-Stadlbauer, Matthias T. Wyss, Aiman S. Saab, Sarah Beggiato, Urs Meyer, Bruno Weber, Tina Notter

**Affiliations:** 1 Institute of Pharmacology and Toxicology, University of Zurich, Zurich, Switzerland; 2 Institute of Veterinary Pharmacology and Toxicology, University of Zurich, Zurich, Switzerland; 3 Department of Life Sciences and Biotechnology, University of Ferrara, Ferrara, Italy; 4 Neuroscience Center Zurich, University and ETH Zurich, Zurich, Switzerland

**Keywords:** Astrocyte, Learning and memory

## Abstract

Astrocytes play critical roles in neural circuit function, but how they contribute to cognitive impairment remains poorly understood. Here, we identify astrocyte-derived kynurenic acid (KYNA), a neuroactive metabolite known to modulate multiple neurotransmitter receptor systems, including the N-methyl-D-aspartate receptor (NMDA), as a mediator of cognitive dysfunction in the context of aberrant astrocyte activity. Using chemogenetic stimulation, pharmacological rescue, and astrocyte-specific knockdown of kynurenine aminotransferase II (KAT II) in mice, we show that elevated KYNA suppresses parvalbumin-positive interneuron activity in the prefrontal cortex, leading to disinhibition of pyramidal neurons and impairments in cognitive functions linked to cortical activity, including episodic-like and working memory as well as sensorimotor gating. These findings define an astrocyte-KYNA-interneuron axis that controls cortical excitability and cognition, linking glial metabolism to circuit imbalance and cognitive dysfunction with potential relevance to psychiatric and neurological disorders.

## Abstract (web summary)

Astrocytes are increasingly implicated in neural circuit function and brain disorders, yet how altered astrocyte states affect cognition remains unclear. Here, the authors show that astrocyte-derived kynurenic acid impairs cognition in mice by suppressing prefrontal inhibitory interneurons.

## Introduction

Astrocytes are increasingly recognized as active participants in neural circuit dynamics. In addition to their classical roles in metabolic and homeostatic support, they modulate synaptic transmission, shape network activity, and influence behavioral states^1,2^. Astrocyte physiology is markedly altered in many neurological and psychiatric conditions^3^. Inflammatory and disease-associated environmental stimuli trigger changes in astrocytic gene expression, intracellular signaling, and metabolic output. These reactive states have been observed in disorders ranging from epilepsy and neurodegeneration to schizophrenia and depression^4,5^.

Despite growing recognition that astrocytes are affected in central nervous system (CNS) disorders, the consequences of these altered states for circuit function and cognition remain poorly understood. This question is especially pertinent to the prefrontal cortex (PFC), a region central to higher-order cognitive function, where circuit dysregulation is strongly linked to cognitive deficits in psychiatric disorders^6^. Cortical circuits in the PFC depend on the precise balance of excitatory and inhibitory activity to sustain working memory, attention, and sensorimotor integration^7^. Maintenance of this balance depends on the coordinated interaction between inhibitory interneurons and pyramidal cells, with fast-spiking parvalbumin-positive (PV+) interneurons and their N-methyl-D-aspartate receptor (NMDAR) activity playing a key role^7,8^. However, it remains unknown whether aberrant prefrontal astrocyte activity alters inhibitory-excitatory circuit interactions and thereby contributes to the emergence of cognitive deficits.

Here, we identify astrocyte-derived kynurenic acid (KYNA), a neuroactive metabolite known to modulate multiple neurotransmitter receptor systems^9^, including the N-methyl-D-aspartate receptor (NMDAR)^10^, as a mediator linking aberrant astrocyte activity to circuit dysfunction and cognitive impairment. Using chemogenetic stimulation, pharmacological rescue, and astrocyte-specific knockdown of kynurenine aminotransferase II (KAT II), we demonstrate that elevated KYNA reduces parvalbumin-positive interneuron activity in the PFC, resulting in pyramidal neuron disinhibition and deficits in PFC-dependent cognitive functions, including episodic-like memory, working memory, and pre-attentive filtering.

## Results

### Astrocyte stimulation in the PFC elevates KYNA and alters cortical circuit activity

**Fig. 1:**
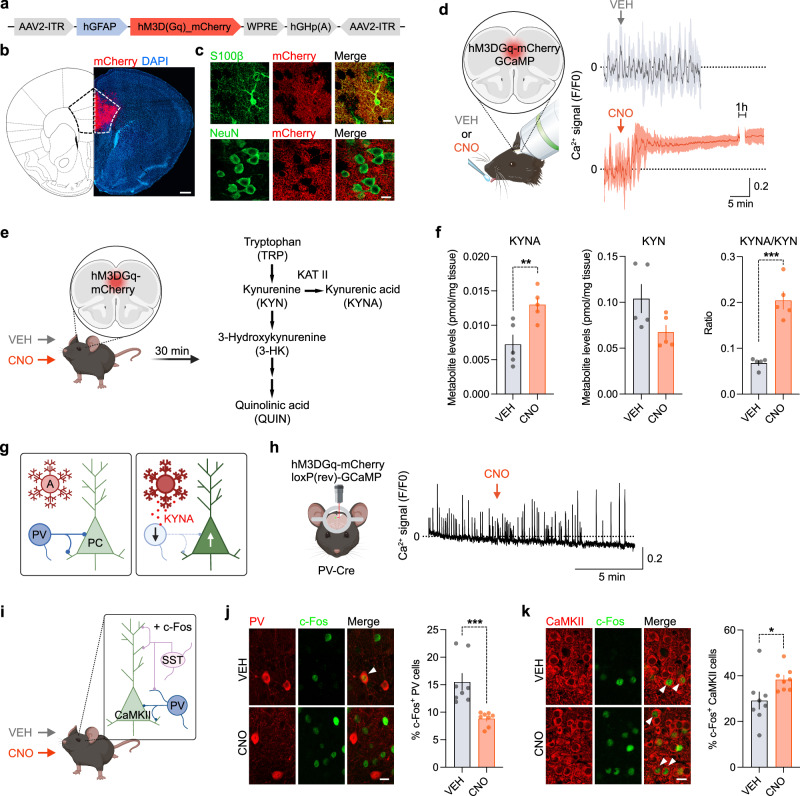
Astrocyte stimulation increases KYNA and shifts PFC circuit dynamics. **a** Simplified scheme of the AAV. **b** Expression of the DREADD construct in the target region of interest (medial portion of the prefrontal cortex (PFC)) after bilateral stereotaxic injection. Image is representative of similar results observed in 80 animals. Scale bar = 500 μm. **c** Representative stains confirming cell type selectivity of construct expression. Astrocytes were stained for S100β (green) and neurons for NeuN (green). Images are representative of similar results observed in 20 animals. Scale bar = 10 μm. **d** In vivo two-photon Ca^2+^ imaging of prefrontal astrocytes in awake mice. Imaging was performed at baseline and after oral administration of either vehicle (VEH) or clozapine-N-oxid (CNO, 1 mg/kg) (Created in BioRender. Notter, T. (2026) https://BioRender.com/q6gg367). Ca^2+^ responses (means ± SD) were indexed by the F/F0 ratio. **e** Experimental setup and simplified kynurenine (KYN) pathway with relevant metabolites (Created in BioRender. Notter, T. (2026) https://BioRender.com/7ochx22). **f** Metabolite levels and ratio in the PFC after VEH or CNO treatment. Each data point represents the pooled samples of two mice (experimental unit). The sample size for each group was *n* = 5. **p = 0.0096, *t*_(8)_ = 3.38 and ***p = 0.0001, *t*_(8)_ = 6.91 (two-tailed). **g** Schematic illustration of the working hypothesis, in which an astrocyte (A)-induced release of kynurenic acid (KYNA) attenuates parvalbumin (PV) interneuron activity (blue), thereby disinhibiting prefrontal pyramidal cell (PC) activity. **h** In vivo two-photon Ca^2+^ imaging of prefrontal PV interneurons (Created in BioRender. Notter, T. (2026) https://BioRender.com/qtvbt0n). Imaging was performed at baseline and after astrocyte stimulation with CNO (1 mg/kg). Ca^2+^ responses were indexed by the F/F0 ratio. **i** Schematic illustration of cell-type-specific c-Fos mapping after astrocyte stimulation (Created in BioRender. Notter, T. (2026) https://BioRender.com/kgjvyev). SST = somatostatin, CaMKII = calmodulin-dependent protein kinase II. **j** PV (red) and c-Fos (green) expression in the PFC after VEH or CNO treatment; arrowhead denotes a c-Fos-positive PV interneuron. Scale bar = 15 μm. The bar plot depicts the % of c-Fos positive PV cells, with each data point representing one animal (experimental unit). The sample size for each group was *n* = 8. ***p = 0.0009, *t*_(14)_ = 4.19 (two-tailed). **k** CaMKII (red) and c-Fos (green) expression in the PFC after VEH or CNO treatment; arrowheads denote c-Fos positive pyramidal cells. Scale bar = 15 μm. The bar plot depicts the % of c-Fos positive CaMKII pyramidal cells after VEH or CNO treatment, with each data point representing one animal (experimental unit). *p < 0.0429, *t*_(14)_ = 2.23 (two-tailed). Scale bars = 15 μm. Data are means ± SEM with individual values overlaid. Source data are provided as a Source Data file.

To examine how altered astrocyte states impact PFC circuit function, we selectively increased astrocyte activity using chemogenetics. To this end, Gq-signaling was activated in PFC astrocytes of adult mice (Fig. 1a–c). In vivo two-photon Ca^2+^ imaging in awake mice confirmed robust and repeatable clozapine-*N*-oxide (CNO)-induced Ca^2+^ elevation in transduced astrocytes (Fig. 1d, Supplementary Fig. S1). Using this approach, we then examined whether altering PFC astrocyte states shifts the metabolic pathway of kynurenine (KYN) degradation. We focused on this pathway (Fig. 1e) because its downstream metabolite, KYNA, is a pleiotropic neuromodulator known to act on multiple neurotransmitter receptor systems, including antagonism of ionotropic NMDA receptors and α7 nicotinic acetylcholine receptors (α7-nAChRs)^9^. KYNA has also been implicated in cognitive dysfunction and is predominantly synthesized by astrocytes^11,12^. Here, we found that PFC KYNA levels and the KYNA/KYN ratio were significantly elevated in CNO-treated mice, while KYN levels and other pathway metabolites remained unchanged (Fig. 1f, Suppl. Fig. S2). These findings indicate that astrocyte stimulation selectively enhances KYNA production in the PFC without altering other KYN pathway metabolites.

According to the excitatory-inhibitory (E/I) imbalance hypothesis, NMDA receptor antagonists, such as ketamine, disrupt prefrontal circuit function by suppressing parvalbumin-positive (PV+) interneuron activity, leading to disinhibition of pyramidal neurons and cognitive impairment^13–15^. Since KYNA is an endogenous NMDAR antagonist^10^, we hypothesized that astrocyte-induced elevated KYNA may similarly reduce PV+ interneuron activity and thereby shift prefrontal circuit dynamics toward excitation (Fig. 1g). To test this, we combined in vivo two-photon Ca^2+^ imaging in PV+ interneurons (Fig. 1h) with cell-type-specific c-Fos mapping (Fig. 1i) following chemogenetic activation of prefrontal astrocytes. In line with our hypothesis, CNO treatment reduced PV+ interneuron activity as evident by the reduction in spike activity (Fig. 1h) and c-Fos expression in PV+ interneurons (Fig. 1j, Suppl. Fig. S3a), while increasing c-Fos in CaMKII+ excitatory pyramidal neurons (Fig. 1k, Suppl. Fig. S3b). No changes were observed in somatostatin-positive (SST+) interneurons (Suppl. Fig. S3c), indicating a selective effect on PV+ cells. Together, these findings demonstrate that altering PFC astrocyte states increases KYNA and suppresses PV+ interneuron activity, leading to disinhibition of pyramidal neurons. This shift in circuit dynamics could provide the mechanistic basis through which astrocytes could contribute to cognitive impairment.

### Astrocyte stimulation impairs memory and pre-attentive filtering

**Fig. 2:**
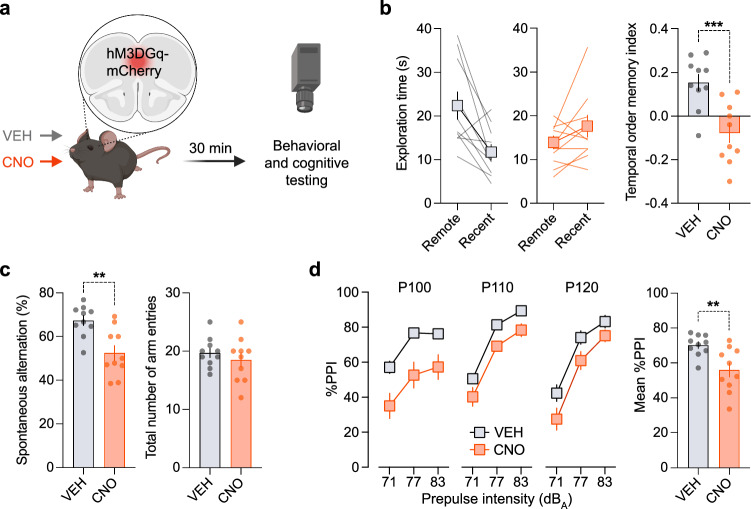
Astrocyte stimulation in PFC impairs cognition and pre-attentive filtering. **a** Scheme of experimental setup (Created in BioRender. Notter, T. (2026) https://BioRender.com/djsd5lb). **b** Absolute exploration times of the temporally remote and recent objects (line plots) and temporal order memory index (bar plot) in the temporal order memory test for objects. ***p = 0.0008, *t*_(18)_ = 4.01 (two-tailed). **c** Percent spontaneous alternation and number of arm entries in the Y-maze test of working memory. **p = 0.002, *t*_(18)_ = 3.61 (two-tailed). **d** Prepulse inhibition (PPI) test of pre-attentive filtering. The line plots show % PPI as a function of prepulse intensity (71, 77 and 83 dB_A_) for each of the three pulse conditions (P100, P110 and P120, which correspond to pulse intensities of 100, 110 and 120 dB_A_). The bar plot depicts the mean % PPI across all prepulse and pulse intensities. **p = 0.005, reflecting the significant main effect of treatment (*F*_(1,18)_ = 9.98) in the full-factorial 2 × 3 × 3 (treatment × prepulse × pulse) repeated-measures ANOVA. All data are means ± SEM with individual values overlaid. Each data point represents one animal (experimental unit). The sample size for each group was *n* = 10. Source data are provided as a Source Data file.

To assess whether astrocyte-induced changes in KYNA and circuit dynamic in the PFC translate into cognitive dysfunctions, we subjected mice to different behavioral and cognitive tests following CNO administration (Fig. 2a). Chemogenetic activation of prefrontal astrocytes impaired performance across several PFC-dependent cognitive domains. In the temporal order memory test for episodic-like memory, CNO-treated male mice failed to discriminate object recency compared to vehicle (VEH) treatment (Fig. 2b). In the Y-maze test for working memory, astrocyte stimulation reduced spontaneous alternation without affecting total arm entries (Fig. 2c). These effects occurred without changes in anxiety-like behavior or locomotion (Suppl. Fig. S4), demonstrating selective effects of astrocyte activation on cognitive performance. Furthermore, CNO-treatment led to the disruption of prepulse inhibition (PPI) of the acoustic startle reflex (Fig. 2d), a form of pre-attentive filtering deficient in various psychiatric disorders^16^ and impaired by elevated endogenous brain KYNA^17^. Control mice expressing a GFAP-driven EGFP construct showed no behavioral effects of CNO (Suppl. Fig. S5), ruling out off-target drug effects.

Behavioral and cognitive analyses in female mice showed that hM3DGq-expressing females displayed similar deficits following CNO treatment (Suppl. Fig. S6), indicating that astrocyte-induced cognitive disruption occurs in both male and female mice. To formally assess potential sex differences, we performed additional full-factorial ANOVAs including sex as an independent factor (Suppl. Table S1). With the exception of PPI, there were no significant sex × treatment interactions in any other behavioral or cognitive test (Fig. 2, Suppl. Fig. S6, Suppl. Table S1). In the PPI test, a significant pulse × treatment × sex interaction was observed (Suppl. Table S1), reflecting that females exhibited a significant deficit only under the 120-dB_A_ pulse condition (Suppl. Fig. S6), whereas males showed an overall significant reduction in PPI across conditions (Fig. 2). Hence, although females displayed a more restricted pattern of PPI impairment, both sexes exhibited treatment-related deficits in pre-attentive filtering, temporal order memory, and working memory.

Together, these findings demonstrate that selective stimulation of prefrontal astrocytes impairs cognitive functions and pre-attentive filtering. The shift towards increased KYNA may underlie the observed changes in circuit activity and behavior, warranting further testing of causality.

### KYNA mediates astrocyte-induced cognitive deficits via PV interneuron suppression

**Fig. 3:**
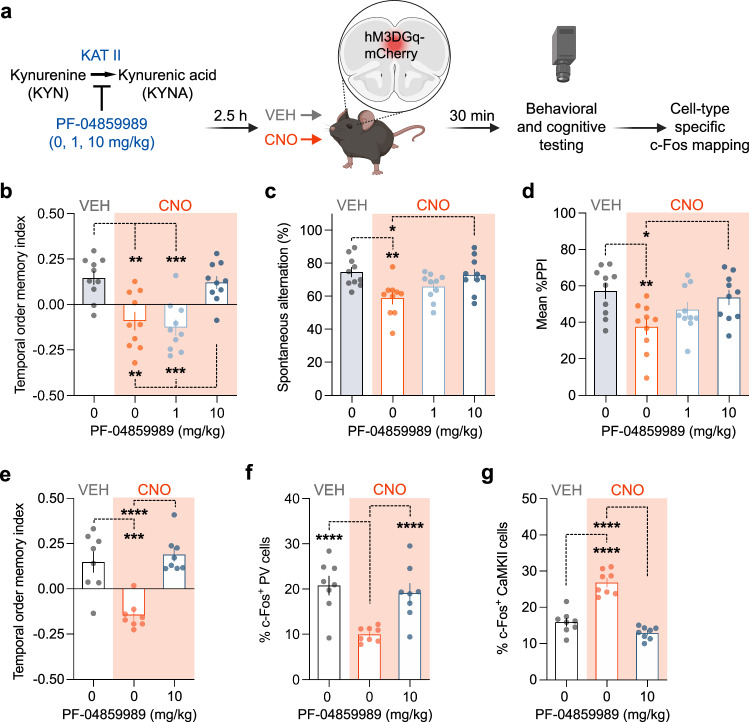
Inhibition of KYNA synthesis reverses cognitive impairments and normalizes PFC cortical dynamics. **a** Schematic representation of the pharmacological rescue study. hM3DGq-expressing mice were pretreated with the kynurenine aminotransferase II (KAT II) inhibitor PF-04859989 (0, 1, or 10 mg/kg, i.p.) 2.5 h before receiving clozapine-N-oxid (CNO, 1 mg/kg) or vehicle (VEH), and 3 h before they were subjected to behavioral and cognitive testing (Created in BioRender. Notter, T. (2026) https://BioRender.com/djsd5lb). **b** Temporal order memory test for objects. **p = 0.0012 and 0.004 and ***p = 0.0002 and 0.0007, based on Tukey’s post-hoc test following one-way ANOVA (*F*_(3,36)_ = 11.96, *p* < 0.0001). **c** Y-maze test of working memory. *p = 0.0123 and **p = 0.0043, based on Tukey’s post-hoc test following one-way ANOVA (*F*_(3,36)_ = 5.60, *p* = 0.0029). **d** Prepulse inhibition test. *p = 0.0415 and **p = 0.0087, based on Tukey’s post-hoc test following one-way ANOVA (*F*_(3,36)_ = 4.46, *p* = 0.0092). **e** Temporal order memory test in hM3DGq-expressing mice pretreated with 0 or 10 mg/kg PF-04859989 2.5 h before receiving CNO or VEH. ***p = 0.0002 and ****p < 0.0001, based on Tukey’s post-hoc test following one-way ANOVA (*F*_(2,21)_ = 19.43, *p* < 0.0001). 2 h after cognitive testing tissue was collected for cell-type-specific c-Fos mapping. **f** % of c-Fos-positive parvalbumin (PV) interneurons. **p = 0.0037 and ***p = 0.0008, based on Tukey’s post-hoc test following one-way ANOVA (*F*_(2,21)_ = 11.04, *p* = 0.0005). **g** % of c-Fos-positive calmodulin-dependent protein kinase II (CaMKII) pyramidal cells. ****p < 0.0001, based on Tukey’s post-hoc test following one-way ANOVA (*F*_(2,21)_ = 54.56, *p* < 0.0001). All data are means ± SEM with individual values overlaid. Each data point represents one animal (experimental unit). The sample size for each group was (**b–d**): *n* = 10 and (**e–g**): *n* = 8. Source data are provided as a Source Data file.

To test whether elevated KYNA causally mediates the cognitive impairments and circuit alterations, we first pharmacologically inhibited its synthesis. We used PF-04859989, a brain-penetrant inhibitor of KAT II, which efficiently lowers brain KYNA levels after systemic administration^18^. Mice received PF-04859989 (1 mg/kg or 10 mg/kg) 2.5 hrs before CNO-induced astrocyte activation (Fig. 3a). While sub-threshold dose (1 mg/kg) had no effect, the higher dose (10 mg/kg) fully restored KYNA levels, temporal order memory, working memory, and PPI in CNO-treated mice (Fig. 3b–d, Suppl. Fig. S7a). These effects were not due to general cognitive enhancement. When the same doses of PF-04859989 were administered to non-activated control mice, no significant changes in cognitive performance were observed (Suppl. Fig. S7b-e). Thus, KAT II inhibition selectively reversed the cognitive deficits induced by astrocyte activation, without baseline pro-cognitive effects. We next examined whether KYNA mediates the shift in cortical circuit dynamics. Administration of 10 mg/kg PF-04859989 prior to CNO treatment restored temporal order memory performance (Fig. 3e) and normalized c-Fos levels in both PV+ interneurons (Fig. 3f, Suppl. Fig. S8a) and CaMKII+ pyramidal neurons (Fig. 3g, Suppl. Fig. S8b), without affecting SST+ interneurons (Suppl. Fig. S8c). To determine whether phenotype magnitude scaled with astrocyte activation, we correlated c-Fos expression in astrocytes and GFAP intensity with cognitive performance and neuronal activity following hM3DGq activation. Consistent with previous work^19^, CNO robustly increased astrocytic c-Fos expression (Suppl. Fig. S9b). In addition, we observed a concomitant increase in GFAP intensity (Suppl. Fig. S9c) that positively correlated with % c-Fos positive astrocytes (Suppl. Fig. S9d), indicating that heightened astrocyte activation is reflected by increased GFAP expression. These increases were independent of PF-04859989 treatment, suggesting that KAT II inhibition does not alter hM3DGq-induced astrocyte activation per se. Consequently, the restoration of cognitive performance and neuronal activity following PF-04859989 treatment reflects a direct effect of KAT II blockade rather than reduced astrocyte activation. Astrocyte activation negatively correlated with cognitive performance (Suppl. Fig. S9e, h) and PV+ interneuron activity (Suppl. Fig. S9f, i), but positively with CaMKII neuron activity (Suppl. Fig. S9g, j), indicating that the degree of astrocyte activation parallels the magnitude of behavioral and circuit-level outcomes.

**Fig. 4:**
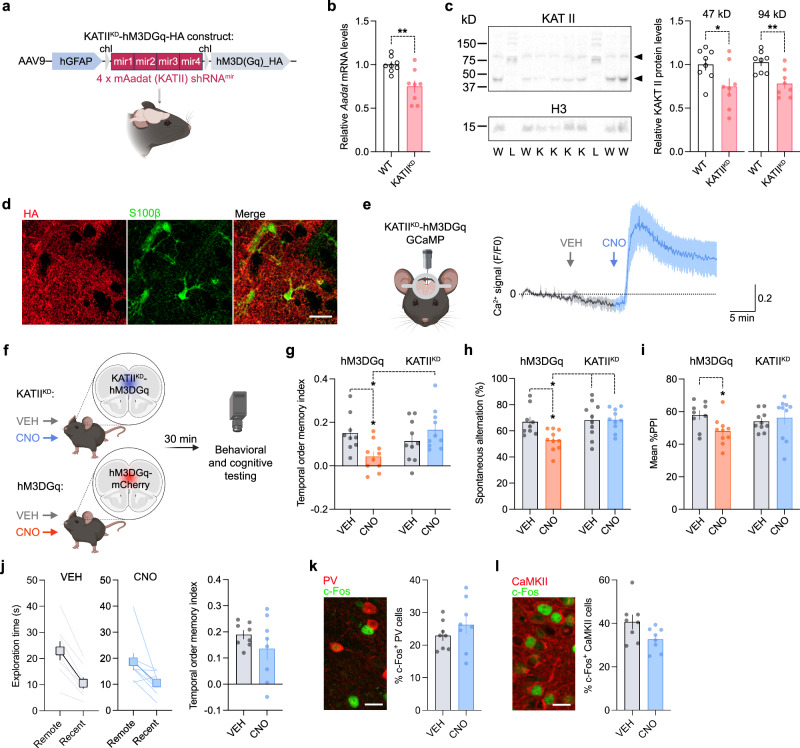
KAT II knockdown prevents astrocyte-mediated cognitive and neuronal deficits. **a** Simplified scheme of the AAV (Created in BioRender. Notter, T. (2026) https://BioRender.com/hvljged). **b** *Aadat* mRNA expression in bulk PFC tissue from KATII^KD^ or wild-type (WT) mice. **p = 0.0039, *t*_(14)_ = 3.45 (two-tailed). **c** Representative KAT II Western blot with PFC lysates from WT (W) and KATII^KD^ (K) mice. Protein ladder (L) and histone 3 (H3) as loading control. Arrows indicate KAT II monomer and homodimer bands. KAT II monomer (left) and KAT II dimer (right) protein levels. *p = 0.0383, *t*_(14)_ = 2.29; **p = 0.0029, *t*_(14)_ = 3.59 (two-tailed). **d** Representative image confirming cell type selectivity of construct expression (stained for HA tag, red and S100β, green). Images are representative of similar results observed in 16 animals. Scale bar = 20 μm. **e** In vivo two-photon Ca^2+^ imaging of prefrontal astrocytes in anesthetized animals (Created in BioRender. Notter, T. (2026) https://BioRender.com/qtvbt0n). Ca^2+^ responses (F/F0 ratio, means ± SD) were measured at baseline and after vehicle (VEH) and clozapine-N-oxide (CNO, 1 mg/kg) treatment. **f** Scheme of experimental setup (Created in BioRender. Notter, T. (2026) https://BioRender.com/f33cvtq). **g** Temporal order memory test for objects. *p = 0.049 and 0.016, based on Tukey’s post-hoc test following two-way ANOVA with a significant interaction (*F*_(1,35)_ = 8.22, *p* = 0.007). **h** Y-maze test of working memory. *p = 0.0307, 0.0123 and 0.0104, based on Tukey’s post-hoc test following two-way ANOVA with a significant interaction (*F*_(1,35)_ = 4.52, *p* = 0.0406). **i** Prepulse inhibition test. *p = 0.0259, based on Tukey’s post-hoc test following two-way ANOVA with a significant interaction (*F*_(1,35)_ = 4.18, *p* = 0.0485). **j** Absolute exploration times of the temporally remote and recent objects (line plots) and temporal order memory index (bar plot) in the temporal order memory test for objects. **k** Quantification of c-Fos-positive parvalbumin (PV)+ cells after VEH or CNO treatment. **l** Quantification of c-Fos positive calmodulin-dependent protein kinase II (CaMKII) pyramidal cells after VEH or CNO treatment. Scale bars = 15 μm. All data are means ± SEM with individual values overlaid. Each data point represents one animal (experimental unit). The sample size for each group was (**b**,**c**): *n* = 8, (**e**): *n* = 3, (**g–i**): *n* = 10 and *n* = 9, (**j–l**): *n* = 8. Source data are provided as a Source Data file.

To further validate the role of astrocyte-derived KYNA in driving these dysfunctions, we next used a genetic approach to block KYNA synthesis selectively in activated prefrontal astrocytes. We hereby injected custom-made AAVs expressing four unique miRNA-adapted shRNAs targeting *mAadat* (referred to as KATII^KD^) along with hM3DGq and an HA tag into the PFC of male mice (Fig. 4a). This design enabled simultaneous chemogenetic activation and selective knockdown of KAT II in prefrontal astrocytes. Knockdown efficacy was confirmed by reduced *mAadat* mRNA and decreased protein levels of both the ~47 kDa KAT II monomer and its active ~94 kDa homodimer^20^ in PFC homogenates of KATII^KD^ mice compared to wild-type controls (Fig. 4b, c). Immunohistochemistry confirmed astrocyte-specific expression in the PFC, as observed with the original hM3DGq-mCherry construct (Fig. 4d). In vivo two-photon Ca²⁺ imaging in anesthetized mice demonstrated that hM3DGq-mediated astrocyte activation remained functional in the KATII^KD^ condition, as indicated by CNO-induced Ca²⁺ elevations (Fig. 4e).

We next assessed whether reducing KAT II expression was sufficient to prevent astrocyte-induced cognitive dysfunction (Fig. 4f). KAT II knockdown prevented the emergence of episodic-like memory (Fig. 4g) and working memory deficits (Fig. 4h), as well as the disruption of PPI of the acoustic startle reflex (Fig. 4i). A concurrent normalization was observed on the level of PV+ interneurons and pyramidal cell activity. Mice expressing KATII^KD^-hM3DGq-HA that failed to show any deficits in temporal order memory (Fig. 4j), working memory (Suppl. Fig. S10a), or PPI (Suppl. Fig. S10b) upon CNO treatment, also displayed unchanged levels of c-Fos expression in both PV+ and CaMKII+ neurons compared to controls (Fig. 4k, l; Suppl. Fig. S10c, d). While the relationship between individual KYNA levels and the magnitude of cognitive deficits remains to be established, these findings suggest that circuit alterations and cognitive impairments are dependent on astrocyte-derived KYNA. Together, these findings identify an astrocyte-KYNA-interneuron axis, whereby astrocyte-derived KYNA impair cognition via impeding PFC E/I balance by selectively suppressing PV+ interneuron activity, leading to disinhibition of pyramidal neurons.

### KYNA contributes to cognitive dysfunction in a neuropsychiatric risk model

**Fig. 5:**
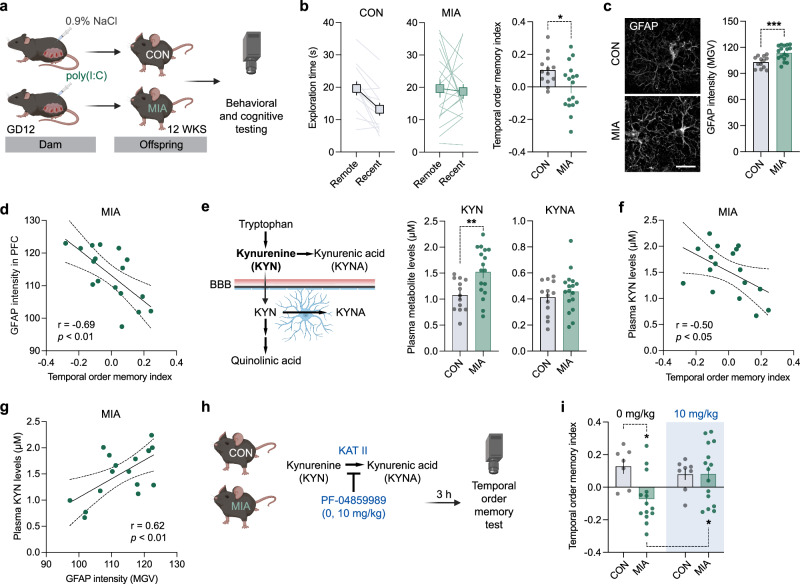
Inhibiting KYNA synthesis reverses MIA-induced cognitive deficits. **a** Pregnant mice were exposed to MIA, induced by poly(I:C) administration, or vehicle (CON) on gestation day (GD) 12. The resulting male offspring underwent behavioral and cognitive testing in adulthood (postnatal week 12 (12 WKS)) (Created in BioRender. Notter, T. (2026) https://BioRender.com/mc6p652). **b** Absolute exploration time of the temporally remote and recent objects (line plots) and temporal order memory index (bar plot) in the temporal order memory test for objects. *p = 0.0272, *t*_(28)_ = 2.33 (two-tailed). **c** Representative stains against glial fibrillary acidic protein (GFAP) in the prefrontal cortex (PFC) and corresponding mean intensity (mean gray value (MGV)) measured in the PFC. ***p = 0.0008, *t*_(28)_ = 3.77 (two-tailed). Scale bar = 20 μm. **d** Pearson’s product moment correlations between prefrontal GFAP intensity and temporal order memory index in MIA offspring. **e** Simplified scheme of the peripheral kynurenine (KYN) pathway depicting blood-brain-barrier (BBB) permeability of KYN, serving as the precursor of the downstream neuroactive metabolite kynurenic acid (KYNA), (produced in astrocytes, as depicted in blue) and quinolinic acid. Plasma levels of KYN and KYNA. **p = 0.0059, *t*_(28)_ = 2.98 (two-tailed). **f** Pearson’s product moment correlations between plasma KYN levels and temporal order memory index in MIA offspring. **g** Pearson’s product moment correlations between plasma KYN levels and prefrontal GFAP intensity in MIA offspring. **h** Schematic representation of the pharmacological rescue study (Created in BioRender. Notter, T. (2026) https://BioRender.com/lshtqyt). **i** Temporal order memory index for CON or MIA offspring receiving 0 or 10 mg/kg PF-04859989. *p = 0.0287 and 0.0407, based on Tukeys post-hoc test following two-way ANOVA (main effect of prenatal treatment: *F*_(1,40)_ = 4.38, *p* = 0.0427; 2-way interaction: *F*_(1,40)_ = 4.57, *p* = 0.0387). All data are means ± SEM with individual values overlaid. Each data point represents one animal (experimental unit). The sample size for each group was (**b**,**c**,**e**): *n* = 13 (CON) and *n* = 17 (MIA), (**i**): *n* = 7 (CON, 0 mg/kg), *n* = 8 (CON, 10 mg/kg), *n* = 14 (MIA, 0 mg/kg), *n* = 15 (MIA, 10 mg/kg). Source data are provided as a Source Data file.

To examine how altered astrocyte states and kynurenine metabolism relate to cognitive dysfunction within a translationally relevant disease framework, we used a mouse model of maternal immune activation (MIA), an established environmental risk factor for neurodevelopmental disorders with cognitive impairments^21,22^. MIA was induced by prenatal administration of poly(I:C), a viral mimetic that triggers an acute inflammatory response during fetal development (Fig. 5a)^23^. Consistent with previous findings^24^, MIA impaired the ability of offspring to discriminate objects based on the temporal presentation (Fig. 5b), which emerged in the absence of concomitant changes in basal locomotor and innate anxiety-like behavior (Suppl. Fig. S11a). We next assessed whether MIA alters astrocyte states in the PFC. GFAP immunoreactivity was significantly elevated in MIA offspring, consistent with increased astrocyte reactivity (Fig. 5c). While other astrocytic markers remained unchanged (Suppl. Fig. S12), individual GFAP levels negatively correlated with cognitive performance in MIA offspring (Fig. 5d), linking astrocyte reactivity to cognitive impairment. Additional partial correlations across the full dataset, controlling for prenatal treatment, consistently revealed a negative correlation between GFAP intensity and temporal order memory (Suppl. Fig. S11b).

We then investigated whether these behavioral and glial changes were accompanied by alterations in KYN metabolism. Compared to controls, MIA offspring displayed elevated plasma KYN levels, the blood brain barrier (BBB)-permeable and immediate precursor of central KYNA^17^, without changes in peripheral BBB-impermeable KYNA (Fig. 5e). Notably, KYN levels negatively correlated with memory performance (Fig. 5f) and positively with prefrontal GFAP intensity (Fig. 5g) in MIA offspring, highlighting a relationship between systemic KYN, astrocyte reactivity, and cognitive deficits under these prenatal conditions. Consistent with this notion, partial correlation analyses across the full dataset identified similar associations among these measures (Suppl. Fig. S11c, d), indicating that systemic KYN levels are generally associated with astrocyte activation and cognitive outcomes beyond the pathological context of MIA.

To test whether elevated KYNA contributes causally to cognitive impairments, adult MIA and control offspring were treated with PF-04859989 (10 mg/kg) or vehicle before testing (Fig. 5h). In MIA offspring, PF-04859989 restored temporal order memory to control levels (Fig. 5i). This effect was not attributable to a general enhancement of cognitive performance, as PF-04859989 treatment had no impact in control offspring, indicating specificity to the pathophysiological state. Together, these findings link heightened prefrontal astrocyte reactivity and KYNA to impaired episodic-like memory performance in a mouse model of psychiatric disease risk, supporting an astrocyte-KYNA contribution to cognitive dysfunction under pathophysiological conditions.

## Discussion

Our study reveals an astrocyte-interneuron signaling mechanism that shapes prefrontal circuit function and cognition. We demonstrate that increased astrocyte activity impairs cognitive performance by elevating the neuroactive metabolite KYNA, which selectively suppresses PV+ interneuron activity and in turn disinhibits pyramidal neurons. The KYNA-driven excitatory-inhibitory imbalance and cognitive deficits are reminiscent of those induced by synthetic NMDAR antagonists such ketamine^13,14,18,25^, PCP^26^, or MK-801^27^. While prior work has implicated astrocyte-derived KYNA in the modulation of excitatory transmission^11^, our study establishes a causal link between astrocyte-driven KYNA elevation, cell-type-specific neuronal effects, and cognitive impairment in vivo. However, additional studies are required to determine whether the observed effects of astrocyte-derived KYNA are mediated through its antagonism of NMDARs^10^ or other neurotransmitter systems, including α7-nAChRs^9^.

Our findings complement recent work on astrocyte-mediated state transitions^28,29^ by identifying a defined astrocyte-KYNA-interneuron axis that disrupts local circuit balance and cognitive performance. Importantly, this axis engages PV+ interneurons, a neuronal population consistently implicated in psychiatric disease pathophysiology^30,31^, and positions astrocytes as upstream modulators of their activity via KYNA. Concurrent to this mechanistic link, our data reinforce the relevance of the KYN pathway in psychiatric diseases. Elevation of prefrontal KYNA levels in the astrocyte DREADD model parallels findings in patients with schizophrenia or bipolar disorder, where increased KYNA has been detected in cortical or cerebrospinal fluid samples^32–36^. Although the exact sources of KYNA in human disease remain unclear, our results suggest that astrocytes are not only capable of producing KYNA under pathophysiological conditions, but also directly contribute to cognitive dysfunction when KYNA levels are elevated.

Alongside our findings, there is a large body of literature demonstrating the importance of astrocytic metabolism and astrocytic-neuronal metabolic coupling in shaping brain activity and cognitive functions^37–39^. For example, sustained astrocytic mitochondrial ROS production modulates brain metabolism and supports cognitive performance^40,41^. KYNA has also been reported to exert antioxidant effects through free radical scavenging, thereby mitigating oxidative stress^42^. Extending this framework, we identify astrocyte-derived KYNA in the PFC as a metabolic factor mediating cognitive dysfunction. Future work will determine whether KYNA primarily disrupts cognition via neurotransmitter receptor antagonism and subsequent cortical disinhibition, or whether it additionally intersects with astrocytic ROS-dependent pathways to shape neural circuit excitability.

Our chemogenetic DREADDs system was based on astrocyte-specific expression of the modified muscarinic Gq-protein-coupled receptor hM3DGq. Activation of Gq proteins mobilizes cytosolic Ca^2+^ via inositol-1,4,5-trisphosphate (IP_3_), a prevalent signaling pathway regulating astrocyte activity^43^. While this system is well suited to probe the functional consequences of aberrant astrocytic activation^19,44^, we did not compare IP_3_/Ca^2+^ signaling with alternative astrocytic pathways, such as astrocytic Gi/Gs signaling. The present study was primarily designed to establish the contribution of astrocytes to KYNA-dependent circuit and behavioral effects rather than to comprehensively dissect the underlying intracellular mechanisms. We therefore consider these findings to provide a strong conceptual and experimental foundation for future studies aimed at systematically distinguishing the relative contributions of these signaling cascades to the identified astrocyte-KYNA-interneuron axis.

We also demonstrate that astrocyte reactivity and KYN metabolism correlate with cognitive deficits in offspring subjected to MIA, a well-established neurodevelopmental model of psychiatric disease risk^21,22^. While MIA induces widespread changes in brain development and processes^22,23^, our data point to a prefrontal astrocyte-KYNA axis as a disease-relevant contributor to cognitive impairments. This is consistent with human studies linking elevated peripheral KYN levels to cognitive deficits in a subset of schizophrenia patients^36^. Moreover, pharmacological inhibition of KAT II, the enzyme converting KYN into KYNA, rescued memory and sensorimotor gating deficits in both MIA and DREADD models, without inducing pro-cognitive effects in control animals. These findings identify KAT II as a potential therapeutic target for conditions marked by glial dysregulation and cognitive dysfunction.

While our study focused specifically on astrocyte activity in the PFC, the astrocyte-KYNA-interneuron signaling axis we define may operate in other brain regions and disease contexts as well. The ability of astrocytes to reshape local circuit dynamics via metabolic modulation of interneuron activity expands our understanding of glial contributions to behavior and identifies a mechanistic link between astrocyte reactivity, KYN metabolism, and cognitive dysfunction. Given the prominence of astrocyte dysregulation, elevated KYNA levels, and PV interneuron deficits across psychiatric disorders such as schizophrenia and bipolar disorder^11,30,32,45–47^, these findings provide a conceptual and mechanistic framework for targeting astrocyte-derived metabolites in the treatment of cognitive symptoms.

## Methods

### Animals

All experiments were performed using male or female C57BL6/N mice (Charles Rivers, Sulzfeld, Germany) or transgenic PV^Cre^ mice (JAX:008069). They were group-housed (4-5 animals per cage) in individually ventilated cages (Allentown Inc., Bussy-Saint-Georges, France). The cages were kept in a specific-pathogen-free (SPF) holding room, which was temperature- and humidity-controlled (21 ± 3 °C, 50 ± 10%) under a reversed light–dark cycle (lights off: 09:00 AM–09.00 PM). All animals had *ad libitum* access to standard rodent chow (Kliba 3336, Kaiseraugst, Switzerland) and water throughout the entire study. All procedures were conducted during the dark cycle. All animal experiments were conducted in accordance with relevant ethical regulations and were approved by the Cantonal Veterinary Office of Zurich (ZH214/2022, ZH028/2023, ZH210/2024, ZH213/2025). All efforts were made to minimize the number of animals used and their suffering. An overview of the different cohorts of animals and the respective numbers used is provided in Table 1. In addition, the number of animals used in each experiment is specified in the legends of the main and Supplementary Figs.

**Table 1:** Overview of the different cohorts of animals and sample sizes used in this study

Cohort	Experimental procedure	Experimental groups	Sample size
1	Awake in vivo two-photon imaging		4
2	Behavioral testing of hM3DGq-expressing male mice	VEH CNO	10 10
3	Behavioral testing of ConV-expressing male mice	VEH CNO	10 10
4	Behavioral testing of hM3DGq-expressing female mice	VEH CNO	10 10
5	Kynurenine metabolite measurement in hM3DGq-expressing male mice	VEH CNO	10 10
6	Behavioral testing of hM3DGq-expressing male mice after pharmacological inhibition of KAT II	0 mg/kg PF-04859989/VEH 0 mg/kg PF-04859989/CNO 1 mg/kg PF-04859989/CNO 10 mg/kg PF-04859989/CNO	10 10 10 10
7	Behavioral testing of C57BL6/N male mice after pharmacological inhibition of KAT II	0 mg/kg PF-04859989 1 mg/kg PF-04859989 10 mg/kg PF-04859989	9 10 10
8	Knockdown verification (mRNA and protein) of KATII^KD^-hM3DGq-expressing male mice and wild-type controls	KATII^KD^-hM3DGq mice Wild-type mice	8 8
9	In vivo two-photon imaging in prefrontal astrocytes expressing KATII^KD^-hM3DGq		3
10	Behavioral testing of KATII^KD^-hM3DGq-expressing and hM3DGq-expressing male mice	VEH, KATII^KD^-hM3DGq CNO, KATII^KD^-hM3DGq VEH, hM3DGq CNO, hM3DGq	10 10 9 10
11	c-Fos expression analyses of behaviorally exposed hM3DGq-expressing male mice	VEH CNO	8 8
12	c-Fos expression analysis of behaviorally tested hM3DGq-expressing male mice after pharmacological inhibition of KAT II	0 mg/kg PF-04859989/VEH 0 mg/kg PF-04859989/CNO 10 mg/kg PF-04859989/CNO	8 8 8
13	Behavioral testing and c-Fos expression analyses of KATII^KD^-hM3DGq-expressing male mice	VEH CNO	8 8
14	Behavioral testing and postmortem analyses in the maternal immune activation (MIA) model	CON MIA	13 17
15	Behavioral testing with pharmacological inhibition of KAT II in the MIA model	CON/0 mg/kg PF-04859989 MIA/0 mg/kg PF-04859989 CON/10 mg/kg PF-04859989 MIA/10 mg/kg PF-04859989	7 14 8 15

### Adeno-associated viruses

The following recombinant adeno-associated viruses (AAVs) were used: AAV9-hGFAP-hM3DGq-mCherry (hM3DGq-mCherry; physical titer: 9.2 × 10E11 vg/mL), AAV9-hGFAP-EGFP (ConV; physical titer: 7.6 × 10E11 vg/mL), AAV9-hGFAP-GCaMP6s (GCaMP6s; physical titer: 3.2 × 10E12 vg/mL), custom made AAV9-hGFAP-chI[4×:sh(mAadat)]-HA_hM3DGq (KATII^KD^-hM3DGq; physical titer: 7.7 × 10E12 vg/mL, see below). The hgfaABC1D GFAP (hGFAP) promoter was used for all constructs to ensure astrocyte-specific expression. For Ca^2+^ imaging of PV interneurons AAV8-hSyn-dlox-jGCaMP8m(rev)-dlox (loxP(rev)-GCaMP; physical titer: 4.2 × 10E12 vg/mL) was injected in transgenic PV^Cre^ mice (JAX:008069). All AAVs were produced and purchased from the Viral Vector Facility of the University of Zurich, Switzerland (www.vvf.uzh.ch).

### DREADD activation

hM3DGq was activated with 1 mg/kg clozapine-N-oxide (CNO, BML-NS105-0025, Enzo Life Sciences, Switzerland) dissolved in 0.9% NaCl (B. Braun, Switzerland). The dose of 1 mg/kg was chosen based on previous chemogenetic studies in rodents^48–51^. Treatment with vehicle (VEH; 0.9% NaCl) served as control treatment. ConV-expressing mice receiving CNO or VEH were used to exclude non-selective effects of CNO^52^. CNO (1 mg/kg) or VEH were given via the micropipette-guided drug administration (MDA) method, a non-invasive oral administration technique described in detail elsewhere^51,53^. Experiments involving in vivo two-photon imaging in anesthetized mice VEH or CNO (1 mg/kg) were injected s.c. with an injection volume of 2 mL/kg. For behavioral testing and tissue collection for brain metabolite measurements, CNO or VEH were given 30 min prior to testing or tissue collection. For c-Fos analyses, CNO or VEH were given either 2 hrs or 5 hrs prior to tissue collection.

### Kynurenine aminotransferase II knockdown strategy and AAV production

To selectively knockdown KAT II expression in astrocytes, we employed the miRNA-adapted short/small hairpin (sh) RNA (shRNAmir) strategy^54,55^. Construct design and AAV production were conducted by the Viral Vector Facility, University of Zurich, Switzerland according to established protocols. shRNA silencing constructs were custom-designed against the *mAadat* transcript NM_011834.2 using the online BLOCK-iT™ RNAi Designer tool (Invitrogen, ThermoFisher Scientific). Four 21-mer shRNA sequences were selected based on their target selectivity (as assessed using the GenScript’s siRNA Target Finder (https://www.genscript.com/tools/sirna-target-finder)) and highest probability ranking for knockdown efficacy. The shRNA sequences were: 5’-GCAACAACCCTACAGGCAACT-3’, 5’-GGTTGAGAGTAGGGTTTATGA-3’, 5’-GGTTTATGACTGGCCCTAAGA-3’, and 5’-GGGTTTCCTGGCTCATATTGA-3’. The four sequences were cloned in silico into the optimized miR-E backbone^55^ and linked, resulting in a chain of 4 hairpins, separated by a spacer sequence. The shRNAmir-E cassette was synthesized using the GeneArt Gene Synthesis service (ThermoFischer Scientific) and cloned into a pssAAV-2-hGFAP-HA_hM3D(Gq)-bGHp(A) backbone, generating the pssAAV-2-hGFAP-chI[4×:sh(mAadat)]-HA_hM3DGq-bGHp(A). The construct was then packaged in AAV9 to produce the AAV9-hGFAP-chI[4×:sh(mAadat)]-HA_hM3DGq (referred to as AAV9-hGFAP-KATII^KD^-hM3DGq-HA or short KATII^KD^-hM3DGq), allowing for region and cell type-selective knockdown of KAT II expression.

Knockdown efficacy was assessed 4 weeks after construct expression by quantitative real-time PCR (qRT-PCR) and Western blot analyses using total RNA and proteins extracted from PFC of male C57BL6/N mice expressing the KATII^KD^-hM3DGq construct in prefrontal astrocytes or C57BL6/N control male mice expressing no construct (referred to as wild-type).

### Stereotaxic surgery

The stereotaxic surgery was performed when the animals reached 10 weeks of age, using methods established and validated before^50,56^. Anesthesia of the animals was induced by inhalation of 4% isoflurane (ZDG9623V, Baxter, Switzerland) in oxygen. After anesthesia induction, the heads of the animals were shaved, and vitamin A cream (Bausch & Lomb Swiss AG) was applied on the eyes to avoid dehydration. The animals were injected with the analgesic Temgesic [buprenorphine (0.1 mg/kg, s.c.), Reckitt Benckiser, Switzerland] and fixed into the stereotaxic frame (MTM-3, World Precision Instruments, USA) while kept under constant isoflurane/oxygen flow [1 to 3% isoflurane in oxygen (600 ml/min)]. All animals were kept on a temperature-controlled heating plate (ATC1000, World Precision Instruments, USA) during the entire surgical procedure to avoid anesthesia-induced hypothermia. Prior to incision, local anesthetics were administered subcutaneously at the incision site (50 μl of a mixture of 1 mL lidocaine (10 mg/mL) and 1 mL bupivacaine (5 mg/mL) in 2 mL saline). A longitudinal incision of the skin was made to expose the skull. The skull was cleaned of connective tissue, and the bone above the target area was removed using a micro drill (OmniDrill35, World Precision Instruments, USA) with a rose burr (ø 0.3 mm). Intracerebral injections were performed using a NanoFil needle and syringe (NANOFIL, NF35BV, World Precision Instruments, USA) connected to an automated pump (UMP3T-1, World Precision Instruments, USA).

Mice received a single, bilateral injection of AAV of interest into the medial portion of the prefrontal cortex (PFC, anteroposterior [AP] = +1.8 mm, mediolateral [ML] = ±0.3 mm, dorsoventral [DV] = −1.9 mm). Solutions were injected at an infusion rate of 10 nL/s and a total volume of 600 nL. After insertion of the needle, a small drop of Histoacryl (B. Braun, Switzerland) was applied at the site of injection to avoid reflux of the injected substances. After injection, the needle was kept in place for 5 min to avoid reflux of the substances before retracting it. Incisions were sutured with a surgical thread (G0932078, B. Braun, Switzerland), and the animals were placed in a temperature-controlled chamber (Harvard Apparatus, USA) until full recovery from anesthesia. After the surgery, the animals were placed back in their home cage and closely monitored for three consecutive days after surgery.

### In vivo two-photon Ca^2+^ imaging using microprisms

In vivo Ca^2+^ imaging using two-photon microscopy in awake or anesthetized mice was applied to ascertain the effectiveness and temporal dynamics of hM3DGq-based elevation of Ca^2+^ in prefrontal astrocytes or Ca^2+^ responses in PV interneurons. To this end, hM3DGq or KATII^KD^-hM3DGq expressing AAV was co-injected with an AAV expressing the Ca^2+^ sensor, GCaMP6s (astrocytes) or loxP(rev)jGCaMP8m (PV interneurons), into the PFC of adult C57BL6/N or PV^Cre^ (JAX:008069) mice respectively. To access the PFC, a right-angled microprism attached to a cranial window was implanted into the subdural space within the fissure opposite the site of injection^57^.

#### Microprism cranial window assembly

On the day of the surgery, a custom-made right angle microprism (1.5-mm side length and 1-mm width, S-BSL7, protected aluminum coating on hypotenuse surface to enable internal reflection; Optosigma) was bonded to a circular glass window (3 mm diameter coverslip) using UV curing optical adhesive (Norland #81).

#### Surgery and virus injection

Surgery and AAV injections were performed using methods established and validated before^57,58^. Headplate implantation, craniotomy, AAV injection and microprism implantation were performed under midazolam (5 mg/kg), fentanyl (0.05 mg/kg), and medetomidine (0.5 mg/kg) anesthesia. First, a chrome steel head plate was implanted. In brief, anesthetized animals were fixed in a stereotaxic frame and an incision was made along the midline to expose the skull. The bone was cleaned, and a bonding agent (Prime & Bond) applied to the skull and polymerized with blue light. A round head plate was attached to the exposed bone using light-cured dental cement (Tetric EvoFlow). The skull over the PFC was left exposed for the craniotomy. Next, the craniotomy was performed using a dental drill (rotate, H-4-002). The skull was first thinned and carefully removed in small bone fragments leaving the dura intact. A custom-made micro injector^59^ was used to unilaterally inject a 1:1 mixture of AAV9-hGFAP-hM3DGq-mCherry or AAV9-hGFAP-chI[4×:sh(mAadat)]-HA_hM3DGq together with AAV9-hGFAP-GCaMP6s or AAV8-hSyn-dlox-jGCaMP8m(rev)-dlox (loxP(rev)-GCaMP (injection volume of 600 nL and injection speed of 10 nL/s). AAVs were injected at a depth of 1.2 mm below cortical surface opposing the site of microprism implantation. Next, the microprism was implanted, whereby an incision in the dura along the side of the sinus was created where the microprism was inserted. The microprism was then gently inserted into the subdural space within the fissure so that the prism surface sat flush opposite the hemisphere to be imaged (with the cerebral falx between the microprism surface and PFC cortex to be imaged). The area beneath the microprism (i.e., the medial portion of the PFC of the contralateral site, which was not imaged) was compressed but remained intact. Dental cement was then used to secure the glass cover slip in place.

#### Training for awake in vivo two-photon imaging

One week after surgery, the training of animals for awake imaging commenced. During the first five days, mice were handled, during which their head was manually fixed by holding the head plate by the experimenter. During these five days, the handling and manual fixation times were gradually increased from 5 min handling and no fixation (first day) to 20 min handling and 5 min fixation (fifth day). At the end of each session, a reward in the form of 40% condensed milk was given via MDA. On day 6, the animals were introduced to being head fixed in the awake imaging setup, whereby the animals were allowed to explore the imaging setup with manual fixation by the experimenter. As of day 7, the animals were being gradually head-fixed in the imaging setup. As for the handling and manual fixation, the times during which animals were head fixed to the imaging setup were gradually increased from 5 to 45 min. The animals were being rewarded with 40% condensed milk via MDA during head fixation and after each session.

#### In vivo two-photon imaging in awake mice

Imaging commenced 3 weeks after virus injection and microprism implantation using a custom-built two-photon laser-scanning microscope^60^. A 16× water immersion microscope objective was used (W Plan-Apochromat 16×/1.0 DIC VIS-IR, Zeiss). GCaMP and mCherry were excited at 940 nm and 1100 nm with a Ti:sapphire laser (Mai Tai; Spectra-Physics) with power between 10 and 30 mW. Fluorescence emission was detected with a GaAsP photomultiplier module (Hamamatsu Photonics) fitted with 520/550 nm band pass filter or a 607/670 band pass filter and separated by a 560 nm dichroic mirror (BrightLine; Semrock). The two-photon laser-scanning microscope was controlled by a customized version of “ScanImage” (r3.8.1; Janelia Research Campus). All imaging was performed in head-fixed awake mice on an air-lifted platform. High resolution images (512 × 512 pixels) at a frequency of 0.74 frames per second and averages of 20 frames of every spot were acquired at the start of every imaging session to ensure localization of the imaging spot over multiple sessions. For Ca^2+^ response measurements, images (128 × 128 pixels) were acquired at a frequency of 5.92 frames per second without averaging. For the VEH imaging session, 5 min baseline activity was recorded. After 5 min, the imaging session was briefly stopped to administer VEH via MDA. Imaging was continued immediately after administration ( ~ 10 to 30 s), and activity after VEH was recorded for 20 min. For the CNO imaging session, 5 min baseline activity was recorded, after which imaging was briefly stopped and CNO (1 mg/kg) was administered via MDA and imaging was continued for 30 min thereafter. One hour after termination of the CNO imaging session, the animals were reimaged for 5 min to assess the duration of CNO-induced overactivation of prefrontal astrocytes. For the second imaging session (at least 48 hrs after the first session), 10 min baseline activity was recorded before administration of CNO (1 mg/kg) as described above. Imaging after CNO was continued for 30 min.

#### In vivo two-photon imaging in anesthetized mice

Imaging in anesthetized mice (1-1.5% isoflurane anesthesia) was conducted as described above with some changes. Imaging commenced two (Ca^2+^ response in PV interneurons) or four weeks (Ca^2+^ response in astrocytes expressing KATII^KD^-hM3DGq) after virus injection and microprism implantation. A 16× water immersion microscope objective was used (W Plan-Apochromat 16×/1.0 DIC VIS-IR, Zeiss). GCaMP6s was excited at 940 nm with a Ti:sapphire laser (Mai Tai; Spectra-Physics) with power between 10 and 30 mW. Fluorescence emission was detected with a GaAsP photomultiplier module fitted with 520/550 nm band pass filter and separated by a 560 nm dichroic mirror (BrightLine; Semrock). Imaging was performed in head-fixed mice on a heating map. For Ca^2+^ response measurements in astrocytes: images (512 × 512 pixels) were acquired at a frequency of 0.74 frames per second without averaging. During the first 10 min, baseline activity was recorded. After 10 min, the imaging session was briefly stopped to administer VEH injection (s.c. injection volume 2 mL/kg). Imaging was continued immediately after administration ( ~ 10 to 30 s) and Ca^2+^ activity in response to VEH was recorded for 10 min. After this, the session was briefly stopped again and CNO (1 mg/kg, s.c. at 2 mL/kg injection volume) was administered. Imaging was immediately resumed and continued for 20 min thereafter. Ca^2+^ response measurements in PV+ interneurons: images (128 × 128 pixels) were acquired at a frequency of 11.84 frames per second without averaging. Baseline activity was recorded for 5 min. Imaging session was briefly stopped to administer CNO (1 mg/kg, s.c. at 2 mL/kg injection volume). Imaging was immediately resumed and continued for 10 min thereafter.

#### Quantification and analysis

Image analysis was performed using the ImageJ software. For each field of view, baseline F0 was defined as the average pixel intensity during the baseline activity recordings of the respective session. This baseline was then subtracted from all pixel intensities for the remaining imaging session. The resulting difference was divided by F0 to obtain dF/F0. To correct for intensity changes due to movement, dF/F0 of the GCaMP signal was normalized to dF/F0 of the mCherry signal for each frame.

### Kynurenine aminotransferase II inhibition

Kynurenine aminotransferase II (KAT II) was inhibited with 1 mg/kg or 10 mg/kg PF-04859989 hydrochloride (PZ0250, Sigma-Aldrich), which was dissolved in sterile water and freshly prepared prior to each experiment. For animals subjected to behavioral testing, 1 or 10 mg/kg PF-04859989, or sterile water (vehicle, 0 mg/kg) only, was injected i.p. using an injection volume of 5 mL/kg 3 h prior to each behavioral test or tissue collection for brain metabolite measurements. For postmortem analyses of c-Fos expression, sterile water (vehicle) or 10 mg/kg PF-04859989 was administered 7.5 h prior to tissue collection. The doses and post-injection interval were chosen based on previous dose-response studies in rodents^18,61^.

### Maternal immune activation model

Two independent cohorts of time-pregnant mice were used in this study. The cohorts were generated via on-site breeding under identical experimental conditions. The first cohort (cohort 14) was used for the assessment of cognitive deficits, followed by post-mortem analyses. The second (cohort 15) was used for the pharmacological rescue study of the cognitive deficits using the KAT II inhibitor PF-04859989.

Timed matings were initiated two weeks after animals had acclimated to the facility. Breeding pairs were set up following previously established protocols^62^, and the presence of a vaginal plug was used to confirm mating success, marking gestational day (GD) 0. A pregnancy was considered successful if a dam exhibited both a vaginal plug on GD 0 and a weight increase of at least 3 grams by GD 12.

On GD 12, pregnant females were randomly assigned to receive either an intraperitoneal (i.p.) injection of low molecular weight (LMW) poly(I:C) (InvivoGen, Toulouse, France; cat.#: tlrl-picw) or a control injection of pyrogen-free 0.9% NaCl (B. Braun, Melsungen, Switzerland). All treated animals received the same lot of poly(I:C) (lot. #: PIW-41-05), which had been previously characterized for quality, composition, and immunostimulatory activity^63,64^. Based on dose-response data from earlier work with C57BL6/N mice^63,64^, poly(I:C) was administered at a dose of 10 mg/kg in a volume of 10 mL/kg. Control animals (CON) received an equivalent volume of vehicle. After injection, dams were returned to their home cages and left undisturbed until five days postpartum. For cohort 14, the number of control and MIA-treated dams was 7 and 7, respectively; in cohort 15, these numbers were 4 and 6. A full description of the maternal treatment protocol is available in the MIA model reporting checklist^65^, provided in Suppl. Table S2.

Male offspring born to CON and MIA dams were weaned on postnatal day 21. All males per litter were selected. This led to a group size of *N* = 13 (CON) and *N* = 17 (MIA) in cohort 14 and *N* = 15 (CON) and *N* = 29 (MIA) in cohort 15. Littermates were housed in groups of 2 to 4 per cage. Behavioral testing in both cohorts commenced when the offspring reached 12 weeks of age. A minimum of 1-week resting period was imposed after behavioral testing before the animals were killed and tissue was collected for subsequent post-mortem analyses.

### Behavioral and cognitive testing

Mice undergoing multiple behavioral tests were given a minimum inter-test interval (ITI) of 48 hrs between tests with the exception between open field test and the temporal order memory test. Because the open field test also serves as habituation to the arena for the temporal order memory test, a 24-hrs ITI was implemented between the two tests.

#### Light-dark box test

A light-dark box test was used to measure innate anxiety-like behavior^66^. The apparatus consisted of four identical multi-conditioning boxes (Multi Conditioning System, TSE Systems, Germany), each containing a dark (1 lux) and a bright (100 lux) compartment. The two compartments were separated from each other by a dark plexiglass wall with an integrated, electrically controlled door. To start a trial, each mouse was placed in the dark compartment. After 5 s, the door automatically opened, allowing access to both the dark and bright compartments for 10 min. The measurements collected from this test included the distance moved and time spent in the light compartment.

#### Open field test

A standard open field exploration task served to assess spontaneous locomotor activity and innate anxiety-like behavior^66^. The apparatus consisted of four identical open-field arenas [40 cm (length) by 40 cm (width) by 35 cm (height)] made of white polyvinyl chloride (OCB Systems Ltd., UK). It was positioned in a testing room with diffused lighting ( ~ 30 lux in the center of the arena). A digital camera was mounted above the arena, captured images at a rate of 5 Hz, and transmitted them to a PC running the EthoVision (Noldus Technology, The Netherlands) tracking system. The animals were recorded for 25 min before they were placed back into their home cage. For the purpose of data collection, the arena was conceptually partitioned into two areas: a center zone (measuring 10 cm by 10 cm) in the middle of the area and a peripheral zone occupying the remaining area. The measurements collected from this test included the total distance moved, and the distance moved in the center zone.

#### Temporal order memory test

A temporal order memory test for objects was used to the animals’ capacity to discriminate the relative recency of stimuli^67^. The test apparatus consisted of an open field as described above, with minor modifications (see below). The test procedure consisted of three consecutive phases, which were each separated by 60 min.

##### Sample phase 1

For this phase, a first pair of identical objects (blue aluminum hairspray bottles, 250 ml, 20 cm high) was placed in the open-field arena in opposing corners approximately 5 cm from the walls. To start a trial, the animals were gently placed into the center of the open field and were allowed to freely explore the objects for 10 min. They were then removed from the apparatus again and kept in a holding room for 60 min before the start of the next phase.

##### Sample phase 2

For this phase, a novel pair of identical objects (LEGO Duplo brick pile, 15 cm high) was placed in the open-field arena, thereby allocating them in the same position as the first pair of objects (see above). To start a trial, the animals were gently placed into the center of the open field again and were allowed to freely explore the novel pair of objects for 10 min. They were then removed from the apparatus once more and kept in a holding room for 60 min before the start of the actual test phase.

##### Test phase

In the test phase, the open field was equipped with one object used in sample phase 1 (temporally more remote object) and one object in sample phase 2 (temporally more recent object), with the corner allocation of the objects being counterbalanced across groups. To start the test trial, the animals were placed into the center of the open field and were allowed to freely explore either object for 5 min. For each animal, a temporal order memory index was calculated by the following formula: [(time spent with phase 1 object)/(time spent with phase 1 object + time spent with phase 2 object)] − 0.5. The temporal order memory index was used to compare the animals’ capacity to discriminate the relative recency of stimuli^67^, with values > 0 signifying a capacity to discriminate between the temporally more remote object presented in sample phase 1 and the temporally more recent object presented in sample phase 2.

#### Short-term memory test in the Y-maze

A spontaneous alternation task in the Y-maze was used to assess working memory^68,69^. This task is based on the innate tendency of rodents to explore novel environments, that is, their preference to investigate a new arm of the Y-maze rather than returning to one that was previously visited^68,69^. The apparatus was made of transparent Plexiglas and consisted of three identical arms (50 cm × 9 cm; length × width) surrounded by transparent Plexiglas walls 10 cm in height. The three arms radiated from a central triangle (8 cm on each side) and were spaced 120° from each other. The maze was elevated 90 cm above the floor and was positioned in a dimly lit room. A digital camera was mounted above the Y-maze apparatus. Images were captured at a rate of 5 Hz and transmitted to a PC running the EthoVision tracking system (Noldus Information Technology), which calculated the total distance moved (m) in the Y-maze.

To start the test, the animals were gently placed in the center of the Y maze and allowed to explore freely for 5 min, whereby the number and sequence of arm entries (defined as entry of the whole body into an arm) were observed and recorded by an experimenter who was blinded to the treatment conditions. Alternation was defined as entry into the three arms in any non-repeating order (for example, ABC, BAC, CBA). Working memory was indexed by the percentage alternation score, which was calculated as the total number of alternations divided by the possible alternations given the number of arm entries (total number of arm entries - 2). In addition to the analysis of percentage alternation, the total distance moved was recorded and analyzed to assess general activity during the 5-min testing period.

#### Prepulse inhibition of the acoustic startle reflex

Pre-attentive filtering was assessed using the paradigm of prepulse inhibition (PPI) of the acoustic startle reflex. PPI of the acoustic startle reflex refers to the reduction in startle reaction in response to a startle-eliciting pulse stimulus when it is shortly preceded by a weak prepulse stimulus. The apparatus consisted of four startle chambers for mice (San Diego Instruments, USA) and has been fully described elsewhere^70^. In the demonstration of PPI, the animals were presented with a series of discrete trials comprising a mixture of 4 trial types. These included pulse-alone trials, prepulse-plus-pulse trials, prepulse-alone trials, and no-stimulus trials in which no discrete stimulus other than the constant background noise was presented. The pulse and prepulse stimuli used were in the form of a sudden elevation in broadband white noise level (sustaining for 40 and 20 ms, respectively) from the background (65 dB_A_), with a rise time of 0.2–1.0 ms. In all trials, 3 different intensities of pulse (100, 110, and 120 dB_A_) and 3 intensities of prepulse (71, 77, and 83 dB_A_) were used. The stimulus-onset asynchrony of the prepulse and pulse stimuli on all prepulse-plus-pulse trials was 100 ms (onset-to-onset).

The protocol used for the PPI test was extensively validated before^62,71,72^. A session began with the animals being placed into the Plexiglas enclosure. They were acclimatized to the apparatus for 2 min before the first trial began. The first 6 trials consisted of 6 startle-alone trials; such trials served to habituate and stabilize the animals’ startle response and were not included in the analysis. Subsequently, the animals were presented with 10 blocks of discrete test trials. Each block consisted of the following: 3 pulse-alone trials (100, 110, or 120 dB_A_), 3 prepulse-alone trials 71, 77, and 83 dB_A_), 9 possible combinations of prepulse-plus-pulse trials (3 levels of pulse × 3 levels of prepulse), and one no stimulus trial. The 16 discrete trials within each block were presented in a pseudorandom order, with a variable interval of 15 s on average (ranging from 10 to 20 s). For each of the 3 pulse intensities (100, 110, or 120 dB_A_), PPI was indexed by percent inhibition of the startle response obtained in the pulse-alone trials by the following expression: 100% × [1 - (mean reactivity on prepulse-plus-pulse trials/mean reactivity on pulse-alone trials)], for each animal, and at each of the three possible prepulse intensities. In addition to PPI, reactivity to pulse-alone trials and prepulse-alone trials were also recorded.

### Tissue collection for postmortem analyses

The animals were deeply anesthetized with an overdose of pentobarbital (Esconarkon ad us. vet., Streuli Pharma AG, Switzerland) and transcardially perfused with ice-cold artificial cerebrospinal fluid (pH 7.4)^50,62,73^. The brains were immediately removed from the skull and either frozen on dry ice and stored at −80 °C until further processing or postfixed in 4% PFA for 6 hrs before cryoprotection in 30% sucrose in PBS for 24-48 hrs, freezing on dry ice and storage at −80 °C until further processing.

Plasma from MIA and CON offspring was collected immediately prior to transcardial perfusion. The atrium was incised, and blood was collected into EDTA-coated blood collection tubes to prevent coagulation. Samples were centrifuged at 2000 × g for 10 minutes to separate plasma, which was then aliquoted and stored at −20 °C until further analysis.

### Immunohistochemistry and laser-scanning confocal microscopy

#### Sample collection and processing

The animals were deeply anesthetized with an overdose of pentobarbital (Esconarkon ad us. vet., Streuli Pharma AG, Switzerland) and transcardially perfused with ice-cold artificial cerebrospinal fluid (pH 7.4)^50,62,73^. The brains were immediately removed from the skull and postfixed in 4% PFA for 6 hrs before cryoprotection in 30% sucrose in PBS for 24-48 hrs. The brains were cut coronally with a sliding microtome at 30 μm (eight serial sections) and stored at −20 °C in cryoprotectant solution [50 mM sodium phosphate buffer (pH 7.4) containing 15% glucose and 30% ethylene glycol; Sigma-Aldrich, Switzerland] until further processing.

#### Immunofluorescent staining

Immunofluorescent stainings were performed according to established protocols^50,51,62,73^. Briefly, the brain sections were rinsed in tris buffer (pH 7.4) before incubating with primary antibodies (GFAP, chicken, polyclonal, ab4674, Abcam, UK, 1:2000; S100β, rabbit monoclonal (EP1576Y), ab52642, Abcam, Switzerland, 1:1000; GS, mouse monoclonal, 610518, BD Transduction Laboratories, 1:1000; Cx43, rabbit polyclonal, 3512, Cell Signaling, USA, 1:1000; mCherry, rat monoclonal (16D7), M11217, Invitrogen, Switzerland, 1:1000; Iba1, rabbit polyclonal, 019-19741, Wako Chemicals, USA, 1:2000; NeuN, rabbit monoclonal (EPR12763), ab177487, Abcam, Switzerland, 1:500; c-Fos, rabbit, monoclonal (9F6), 2250, Cell Signaling, USA, 1:1000; CaMKII alpha, mouse, monoclonal (6G9), GeneTex, USA, 1:500; PV, guinea pig, polyclonal, 195 004, Synaptic Systems, Germany, 1:1000; SST, mouse, monoclonal (C11), AA 27-116, Antibodies online, USA, 1:500; HA-tag, goat, polyclonal, A190-138A, Fortis Life Sciences, USA, 1:2000). The primary antibodies were diluted in tris buffer containing 0.2% Triton X-100 and 2% normal serum. The sections were incubated free-floating under constant agitation (100 rpm) overnight at 4 °C. The following day, sections were washed three times for 10 min in tris buffer before a 30-min incubation period with secondary antibodies (Alexa488 (Molecular Probes, Eugene, USA, 1:1000), Cy3 (Jackson ImmunoResearch, UK, 1:500), or Cy5 (Jackson ImmunoResearch, UK, 1:500), and DAPI (1 mg/mL H_2_O; Thermo Fisher Scientific, Switzerland, 1:3000)) diluted in tris buffer containing 2% normal serum at room temperature. After incubation, which was shielded from light, the sections were washed 3 × 10 min in tris buffer, mounted onto gelatinized glass slides, coverslipped with Dako fluorescence mounting medium (S3023, Agilent, Switzerland), and stored in the dark at 4 °C until image acquisition.

#### Image acquisition

Immunofluorescence images were captured by laser scanning confocal microscopy or with Airyscan confocal microscopy (Zeiss LSM800 with Airyscan). To assess the selectivity of hM3DGq construct expression, 6 images randomly selected from 3 consecutive sections within the area of construct expression were acquired per animal using a 40× (oil, NA 1.4) objective with a zoom of 0.45. Each image contained 4 consecutive optical sections (1024 × 1024 pixels, spaced 1 μm in z). Higher resolution image stacks for representative images of cell type specific expression of hM3DGq-mCherry were acquired in Airyscan mode using a 40× lens, NA 1.4, oil and processed using the default settings provided by ZEN 2.6 blue edition software (Zeiss, Switzerland). For the c-Fos expression analyses in different cell types of the PFC and GFAP intensity analyses, 6 randomly selected single plane images (1024 × 1024 pixels) within the area of construct expression across 3 consecutive sections were acquired per animal using a 25× (oil, NA 0.8) objective with a zoom of 1. For the intensity analyses of astrocyte markers (MIA model), 9 images randomly selected from 3 consecutive sections within the PFC were acquired per animal using a 25× (oil, NA 0.8) objective with a zoom of 0.7. Each image contained 3 consecutive optical sections (1024 × 1024 pixels, spaced 1 μm in z). Images for each experiment were acquired on the same experimental day by an experimenter blinded to the experimental conditions, whereby imaging settings were kept constant throughout an entire imaging day. Final illustrations were obtained using ImageJ or Imaris software, with contrast and brightness uniformly adjusted across each entire image.

#### Image analyses

Image analyses were performed using the ImageJ software by an experimenter blinded to the experimental conditions. For the assessment of selective expression of the hM3DGq construct in astrocytes, the number of mCherry + , S100β + , NeuN + , mCherry + /S100β + , and mCherry + /NeuN+ cells were counted within each image. Efficacy of transduction was calculated by dividing the number of colocalized cells (mCerry + /S100β + ) with the number of S100β+ cells and multiplied by 100. Expression selectivity was calculated by dividing the number of colocalized cells with the number of total mCherry+ cells and multiplied by 100. For the cell-type specific c-Fos mapping within the PFC, the number of c-Fos+ cells, the number of target cells, and the number of co-localized cells were counted within each image. The percentage of c-Fos+ cells was calculated as follows: (number of co-localized cells/number of target cells) multiplied by 100 for each image (referred to as field of view (FOV)). The mean % of c-Fos+ cells, the mean number of target cells, and the mean number of c-Fos+ cells were then calculated over the 6 images for each animal. Intensity (mean gray value) per astrocyte marker was measured and calculated on single plane (GFAP intensity in the hM3DGq model) or z-projected images (astrocyte markers in the MIA model) with a threshold applied to remove background. The mean intensity was than calculated over the 6 or 9 images for each animal.

### Quantification of brain metabolites of the kynurenine pathway

Brain metabolites of the kynurenine pathway were measured using liquid chromatography-nanoelectrospray ionization tandem mass spectrometry (LC-NSI-MS/MS) performed at the Functional Genomic Center Zurich (Metabolomics at FGCZ, University of Zurich). The metabolites assessed included kynurenine (KYN), kynurenic acid (KYNA), tryptophan (TRP), quinolinic acid (QUIN), and 3 hydroxykynurenine (3-HK). Plasma KYN and KYNA were measured in CON and MIA offspring using high-performance liquid chromatography (HPLC) with fluorimetric detection (HPLC-FLD).

### Liquid chromatography-nanoelectrospray ionization tandem mass spectrometry

#### Sample collection

Male mice expressing hM3DGq in the PFC were treated orally with 1 mg/kg CNO or 0.9% saline. 30 min after treatment they were deeply anesthetized with an overdose of pentobarbital (Esconarkon ad us. vet., Streuli Pharma AG, Switzerland) and transcardially perfused with ice-cold artificial cerebrospinal fluid (pH 7.4)^50,62,73^ to flush out blood-derived metabolites of the kynurenine pathway. The brains were immediately removed from the skull, and the PFC was dissected on ice as described before^50,56,68^. The PFC samples from two mice were pooled, weighed and homogenized in 200 μL 80% ice-cold methanol using a sterile BioMasher (9791 A, Takara, France). For male mice expressing hM3DGq in the PFC that were pretreated with 0 mg/kg, 1 mg/kg, or 10 mg/kg PF-04859989 2.5 h prior to VEH or CNO treatment and 3 h prior to tissue collection (as described above), PFC sample from one animal was homogenized in 200 μL 80% ice-cold methanol using a sterile BioMasher (9791 A, Takara, France). Tissue weights could not be taken during tissue collection. The samples were frozen with dry ice and transported to the Functional Genomics Center Zurich, University of Zurich for targeted metabolomic analysis.

#### Sample processing

The thawed samples were centrifuged (15 min / 14 krpm / + 4 °C). From each clear, colorless supernatant 180 µL were transferred to 1.5 mL eppendorf tubes, evaporated to dryness under a gentle stream of nitrogen and re-dissolved in 200 µL water containing 10 mM ammonium formate (pH 4.2) and 0.1 % (v/v) formic acid prior to mass spectrometry analysis.

#### LC-NSI-MS/MS analysis

LC-NSI-MS/MS was performed on a TSQ Quantiva triple quadrupole mass spectrometer (ThermoFisher Scientific, United States) coupled to an ACQUITY UPLC M-Class (Waters, United States) system using nanoelectrospray ionization. The analysis was conducted using a capillary column (150 µm ID, 5.5 cm length, 15 µm orifice) created by hand packing a commercially available fused-silica emitter (MSWIL, Netherlands) with HSS T3 separation media (Waters, United States). The mobile phase consisted of 10 mM ammonium formate (pH 4.2) and 0.1 % (v/v) formic acid in water (A1) and 0.1 % (v/v) formic acid in acetonitrile (B1). A 1 µL injection loop was used and the sample (1 µL) was loaded onto the capillary column with 2 µL/min flow at the initial conditions (92.5 % A1, 7.5 % B1) and eluted under isocratic conditions at a flow rate of 2 µL/min over 10 min, following by ramping to 98 % B1 within 1 min and holding at this composition for an additional 4 min. The column was then re-equilibrated at the initial conditions for 5 min before the next injection. The nanoelectrospray source was operated in positive ion mode and the voltage set at 2.7 kV. The ion transfer tube temperature was 250 °C and the radio frequency (RF) lens set at 90 V. The collision gas was Ar at 1.5 mTorr with a collision energy of 14 eV and the quadrupoles were operated at a resolution of 0.4 Da for Q1 and of 0.7 Da for Q3. The mass transitions for monitoring the analytes were m/z 190 → 144.1 for KYNA, 209.1 → 192.2 for KYN, m/z 205.1 → 146.1 for TRP, m/z 168 → 124 for QUIN, and m/z 225 → 162.1 for 3-HK, respectively.

The quantitation of the analytes was done using the mass spectrometers vendor software package Quan Browser in the software suit Xcalibur based on the peak areas and the constructed calibration curves. Calibration curves were constructed for each analyte during each analysis using a series of standard solutions of analytes. Pmole/mg tissue was calculated using the following calculation: ((nM of metabolite measured in injected volume) * 0.001 * 200)/(tissue weight).

### High-performance liquid chromatography with fluorimetric detection

#### Sample collection

The animals were deeply anesthetized with an overdose of pentobarbital (Esconarkon ad us. vet., Streuli Pharma AG, Switzerland). Prior to transcardial perfusion with ice-cold, artificial cerebrospinal fluid (CSF) (pH 7.4) and subsequent brain collection (see above), the atrium was incised, and blood was collected into EDTA-coated blood collection tubes (cat. # 365975, BD, USA) to avoid coagulation. Plasma was separated by centrifugation (2000 × g, 10 min) and stored at −20 °C until further use.

#### Sample processing

Plasma samples for KYN and KYNA measurements were diluted (1:2 v/v and 1:10 v/v, respectively) with ultrapure water to a final volume of 100 µl with. Next, the diluted samples were acidified with 25 µl of 6% perchloric acid. After centrifugation (12,000 × g, 10 min), metabolite levels were measured in 20 µl of the resulting supernatant by HPLC-FLD.

#### HPLC-FLD

20 μL of each processed supernatant was injected onto a 3 μm C18 reversed-phase HPLC column (100 mm × 4 mm; Dr. Maisch GmbH, Ammerbuch, Germany). The mobile phase consisted of 50 mM sodium acetate and 4% acetonitrile (v/v), pH adjusted to 6.2 with glacial acetic acid, and was delivered at a flow rate of 0.5 mL/min. Zinc acetate (0.5 M, unadjusted pH) was introduced post-column using a peristaltic pump (AXP, Dionex/Thermo Fisher Scientific, Waltham, MA, USA) at 0.1 mL/min.

In the eluate, KYN and KYNA were determined by fluorimetric detection using a Jasco FP-2020 Plus spectrofluorometer (Jasco Inc., Tokyo, Japan). Excitation/emission wavelengths were 365/480 nm for KYN and 344/398 nm for KYNA. Under these conditions, retention times of KYN and KYNA were approximately 6 min and 14 min, respectively.

### Quantitative real-time PCR analysis

qRT-PCR was used to measure *mAadat* RNA levels in PFC extracted from adult C57BL6/N mice expressing no construct (referred to as wild-type) and mice that express the KATII^KD^-hM3DGq-HA construct in astrocytes. RNA extraction and quantitative qRT-PCR analyses were performed according to established protocols^50,51,74^. In brief, the animals were deeply anesthetized with an overdose of Nembutal (Abbott 23 Laboratories, North Chicago, IL, USA) and transcardially perfused with 20 ml ice-cold, artificial CSF (pH 7.4). After decapitation, the brains were immediately extracted from the skull, frozen on dry ice, and stored at −80 °C until further processing. One hemisphere was cut into 1-mm coronal brain sections using razorblade cuts and subsequent micro-dissection of the medial portion of the PFC. Total RNA was extracted using the SPLIT RNA extraction kit (Lexogen, Austria) following the manufacturer’s recommendations. Extracted RNA was quantified by Nanodrop (DeNovix DS-11+ spectrophotometer, Labgene Scientific SA, Switzerland) and analyzed by a TaqMan qRT-PCR instrument (CFX384 real-time system, Bio-Rad Laboratories) using the iScript one-step qRT-PCR kit for probes (Bio-Rad Laboratories) as previously described^50,51^. A mouse TaqMan gene expression assay for Aadat (assay ID: Mm00496169_m1, catalog number: 4331182; Thermo Fisher Scientific, Zurich, Switzerland) was used. The samples were run in 384-well formats in triplicates as multiplexed reactions with the normalizing internal control (36B4) as validated previously^50,51^. Relative gene expression was calculated with the 2 − ΔΔCt method. All qRT-PCRs and analyses were conducted by an experimenter blind to the experimental conditions. Relative changes in *mAadat* expression were finally compared to the average relative *mAadat* expression in the PFC of wild-type C57BL6/N mice.

### Western blot analysis

Western blot was used to investigate KAT II protein levels in total PFC homogenates extracted from adult C57BL6/N male mice expressing no construct (referred to as wild-type) and adult male mice that express KATII^KD^-hM3DGq-HA construct in astrocytes. PFC were dissected from one brain hemisphere as described above (see previous paragraph). Lysis and sample preparation were performed as previously described^75,76^, with the following adaptations: Tissue was lysed in ice-cold RIPA buffer with a TissueLyserII (Qiagen, Germany) and centrifuged at 10,000 × g at 4 °C for 30 min. The supernatant was collected, and total protein concentration was measured using the Qubit Protein and Protein Broad Range (BR) Assay Kit according to protocol (Q33211, Thermo Fisher Scientific, Switzerland). Equal amounts of protein (180 μg) were run under reducing conditions on 4-20% Bis-Tris (M00657, GenScript, UK) and then electrophoretically transferred onto PVDF membranes (ISEQ00005, Millipore, USA). The blots were blocked with 5% non-fat dry milk in PBS-T for 1 hr. The membrane was cut and incubated with the respective primary antibodies (AADAT, rabbit, polyclonal, PA5-88974, Invitrogen,Switzerland, 1:700, and Histone 3, rabbit, monoclonal (D1H2), 4499, Cell Signaling, USA, 1:1000) in blocking solution overnight at 4 °C. The membranes were washed and incubated with horse radish peroxidase-conjugated anti-rabbit secondary antibody (anti-rabbit hrp-linked, Cytiva NA934, Millipore, USA) in blocking solution for 1 h at room temperature. After washing the immunocomplexes were visualized by chemiluminescence using the SuperSignal West Pico PLUS Chemiluminescent Substrate (34579, Invitrogen, Switzerland). Images were acquired using the Image Lab™ Upgrade for ChemiDoc™ XRS+ System (1708299, Bio-Rad, USA). Tiff images were analyzed using the Fiji software. Levels of KAT II were analyzed with reference to H3 as the housekeeping control. Each sample was run twice, and the average was used in the final analysis. All analyses were conducted by an experimenter who was blind to the experimental conditions. Levels of KAT II protein in the PFC of KATII^KD^-hM3DGq-HA mice were finally compared to the average KAT II levels in the PFC of wild-type C57BL6/N mice.

### Statistics and Reproducibilty

All behavioral, cognitive, immunohistochemical and molecular data were acquired and analyzed in a blind manner, in which the treatment conditions were blinded in the form of numerical codes. Likewise, all samples collected for HPLC-FLD and LC-NSI-MS/MS analyses were randomly labeled by an experimenter before the measurements and analysis were conducted, with the samples being unblinded once all data were collected and analyzed. All statistical analyses were performed using SPSS Statistics (version 28.0, IBM, Armonk, NY, USA) and Prism (version 10.0; GraphPad Software, La Jolla, CA, USA). Statistical significance was set at *p* < 0.05. All data met the assumptions of normal distribution and equality of variance.

In the temporal order memory test, temporal order memory index was assessed by independent Student’s *t*-test (two-tailed). In the open field test, the total distance moved, and the distance moved in the center zone were assessed using 2 × 5 (treatment × bins) repeated-measure ANOVAs. In the Y-maze test, spontaneous alternation and total arm entries were assessed using independent Student’s *t*-tests (two-tailed). In the PPI test, % PPI and startle reactivity to pulse-alone trials were analyzed using 2 × 3 × 3 (treatment × prepulse intensity × pulse intensity) and 2 × 3 (treatment × pulse intensity) repeated-measure ANOVAs, respectively. In the light-dark box test, the time spent in the light or dark compartment was analyzed by a 2 × 2 (treatment × compartment) repeated-measure ANOVA, whereas the total distance moved was evaluated using independent Student’s *t*-test (two-tailed). Additional full-factorial ANOVAs including sex as an independent factor were conducted for each behavioral test with data from male and female hM3DGq mice. Group differences in the pharmacological rescue study in hM3DGq mice (behavioral data and postmortem analyses) were assessed using one-way ANOVA. Data from the pharmacological rescue study involving the MIA model were assessed using two-way ANOVA. Likewise, behavioral data involving KATII^KD^-hM3DGq- and hM3DGq-expressing mice were assessed using two-way ANOVA. Whenever appropriate, ANOVAs were followed by Tukey’s post-hoc test for multiple comparisons. Group differences in metabolites of the KYN pathway were assessed by independent Student’s *t*-tests (two-tailed). Group differences in the c-Fos analyses were either assessed by independent Student’s *t*-tests (two-tailed) or one-way ANOVA followed by Tukey’s post-hoc test. Correlative analyses in the MIA model were conducted using Pearson’s product moment correlations (MIA offspring) and two-tailed partial correlations across all offspring controlling for prenatal treatment effects. Likewise, correlative analyses involving measures of astrocyte activation, behavioral and cellular outcomes were conducted for CNO-treated hM3DGq mice using Pearson’s product moment correlations. Correlation between different measures of astrocyte activity were conducted for CNO-treated hM3DGq mice using two-tailed partial correlations controlling for the effects of PF-04859989 treatment.

All experiments were independently and successfully replicated, except for the two-photon imaging experiments involving KATII^KD^-hM3DGq or PV interneuron activity and the pharmacological rescue experiments in the MIA model, which were performed once.

### Reporting summary

Further information on research design is available in the Nature Portfolio Reporting Summary linked to this article.

## Supplementary information

Supplementary Information

Reporting Summary

Transparent Peer Review file

## Source data

Source data

## Data Availability

All data needed to evaluate the conclusions in the paper are presented in the main text or Supplementary Information. Source data are provided with this paper.
