# Supplementary material for: Astroglial disinhibition of cortical circuits disrupts cognition via kynurenic acid in mice
Source: Nat Commun. 2026 May 8;17:6210. doi: 10.1038/s41467-026-72640-0 (PMC13369966; doi:10.1038/s41467-026-72640-0)
Supplement: Supplementary file 1 — Supplementary Information [file 41467_2026_72640_MOESM1_ESM.pdf]

SUPPLEMENTARY INFORMATION

Astroglial disinhibition of cortical circuits disrupts cognition via kynurenic acid in mice

Viktor Beilmann[1§], Johanna Furrer[1§], Sina M. Schalbetter[2], Ron Schaer[2], Edoardo Tiziani[3], Kim D. Ferrari[1,4], Felisa Herrero[2], Celine Heeb[2], Alexandra von Faber-Castell[1,4], Jacqueline Condrau[1,4], Ulrike Weber-Stadlbauer[2,4], Matthias T. Wyss[1,4], Aiman S. Saab[1,4], Sarah Beggiato[3], Urs Meyer[2,4], Bruno Weber[1,4], Tina Notter[1,4]*

[1] Institute of Pharmacology and Toxicology, University of Zurich, Zurich, Switzerland.

[2] Institute of Veterinary Pharmacology and Toxicology, University of Zurich, Zurich, Switzerland.

[3] Department of Life Sciences and Biotechnology, University of Ferrara, Ferrara, Italy.

[4] Neuroscience Center Zurich, University and ETH Zurich, Zurich, Switzerland.

§ These authors contributed equally to this study.

Correspondence:

Dr. Tina Notter (PhD)

Institute of Pharmacology and Toxicology, University of Zurich

Winterthurerstrasse 190, CH-8057, Zurich, Switzerland.

E-mail: tina.notter@pharma.uzh.ch

Tel.: +41 44 63 55315

SUPPLEMENTARY DATA

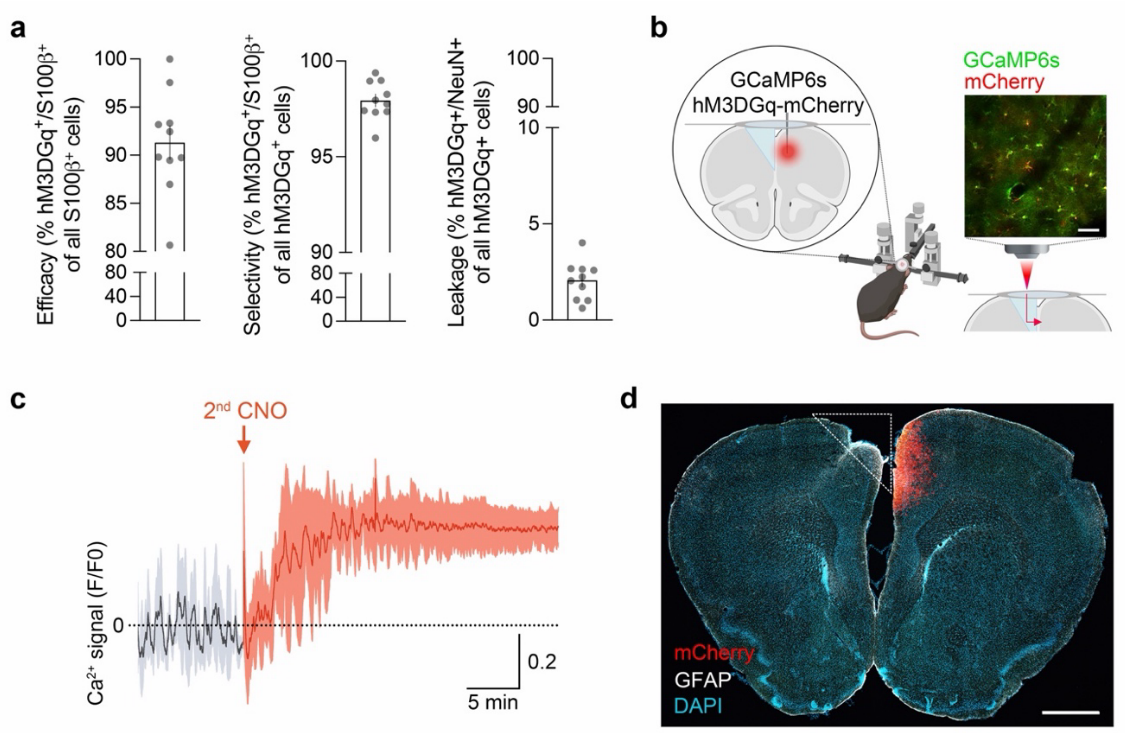

Figure S1. Selectivity and effectiveness of hM3DGq-based stimulation of prefrontal astrocytes. (a) Quantification of the efficacy of transduction, as well as cellular selectivity of hM3DGq-mCherry construct expression in astrocytes (S100β) and neurons (NeuN). Scatter bar plots represent the % of astrocytes that express hM3DGq-mCherry (left plot, efficacy of transduction), the % of hM3DGq-mCherry-positive cells that were astrocytes (middle plot, selectivity), or the % of hM3DGq-mCherry-positive cells that were neurons (right plot, leakage) in the PFC. Each data point represents one animal (experimental unit). The sample size for each group was *n* = 10. **(b)** Simplified scheme of the surgical procedure for subsequent *in vivo* two-photon Ca^{2+} imaging of prefrontal astrocytes (Created in BioRender. Notter, T. (2026) https://BioRender.com/fes827n). The photomicrograph shows a high-resolution image of prefrontal astrocytes co-expressing the hM3DGq-mCherry and GCaMP6s construct acquired by *in vivo* two-photon imaging in awake animals. Scale bar = 25 µm. **(c)** Ca^{2+} response (F/F0 ratio, means ± SD, *n* = 4 mice) measured 48 hrs after the first imaging session and clozapine-N-oxide (CNO, 1 mg/kg) treatment. Baseline Ca^{2+} activity and Ca^{2+} response in astrocytes to a second CNO treatment are comparable to the first CNO treatment (see main *Figure 1*). **(d)** Representative tile scan image of a brain section stained for mCherry (red), GFAP (white), and DAPI (blue) showing the location of microprism implantation (dashed line). Scale bar = 1mm. Source data are provided as a Source Data file.

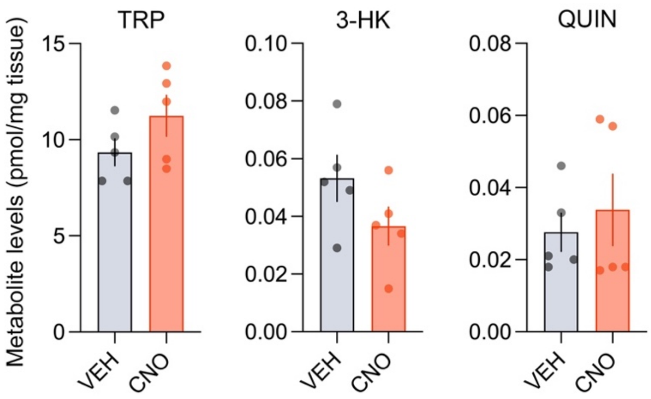

Figure S2. Effects of astrocyte stimulation on prefrontal levels of metabolites of the kynurenine pathway. Male mice expressing hM3DGq in prefrontal astrocytes were treated with vehicle (VEH) or clozapine-N-oxide (CNO, 1 mg/kg) 30 min before collection of brain samples. Levels of tryptophan (TRP), 3-hydroxykynurenine (3-HK), and quinolinic acid (QUIN) in the prefrontal cortex after VEH or CNO treatment. Each data point represents the pooled samples of two mice (experimental unit). The sample size for each group was $n = 5$. Source data are provided as a Source Data file.

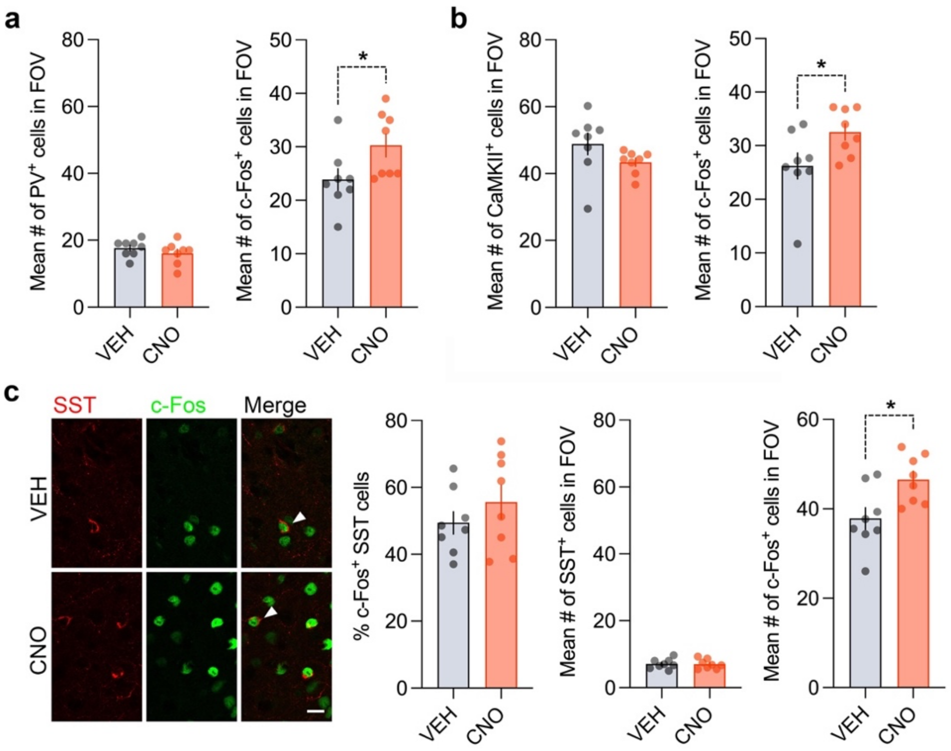

Figure S3. Cell-type-specific c-Fos mapping in the prefrontal cortex following astrocyte stimulation. (a) c-Fos expression in parvalbumin (PV)+ interneurons. The bar plots depict the mean number (#) of PV+ cells (left) and the mean # of c-Fos positive cells (right) in the field of view (FOV). *p < 0.05, $t_{(14)}$ = 2.16 (two-tailed). **(b)** c-Fos expression in calmodulin-dependent protein kinase II (CaMKII)+ excitatory pyramidal cells. The bar plots depict the mean # of CaMKII+ cells (left) and the mean # of c-Fos positive cells the mean (right) in the FOV. *p < 0.05, $t_{(14)}$ = 2.22 (two-tailed). **(c)** c-Fos expression in somatostatin (SST)+ interneurons. SST (red) and c-Fos (green) expression in the prefrontal cortex after vehicle (VEH) or clozapine-N-oxide (CNO, 1 mg/kg) treatment; arrowhead denotes c-Fos-positive SST interneurons. Scale bar = 25 µm. The bar plots depict the % of c-Fos positive SST cells (left), the mean # of SST+ cells in the FOV (middle), and the mean # of c-Fos positive cells in the FOV. *p < 0.05, $t_{(14)}$ = 2.79, (two-tailed). All data are means ± SEM with individual values overlaid. Each data point represents one animal (experimental unit). The sample size for each group was n = 8. Source data are provided as a Source Data file.

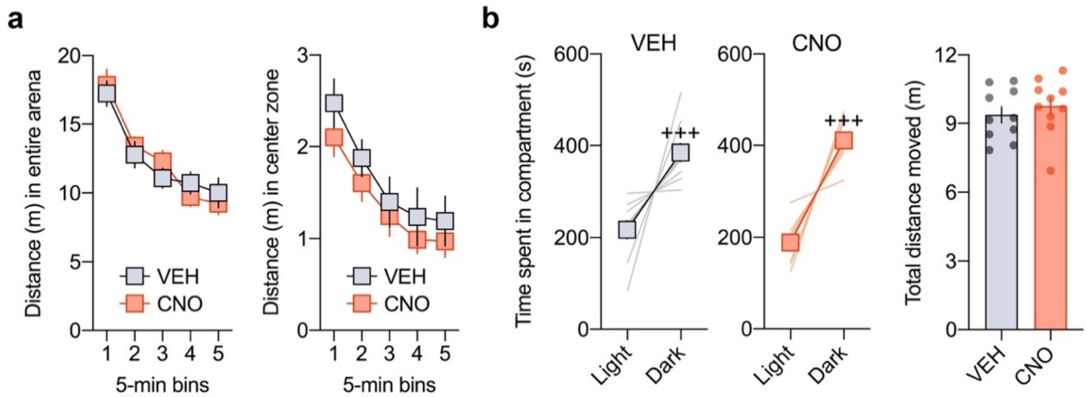

Figure S4. Performance in the open field and light-dark box tests after chemogenetic activation of prefrontal astrocytes. Male mice expressing hM3DGq in prefrontal astrocytes were treated with vehicle (VEH) or clozapine-N-oxide (CNO, 1 mg/kg) and were then subjected to behavioral and cognitive testing 30 min after treatment. **(a)** Distance moved as a function of 5-min bins in the entire arena (left) and center zone (right) during the open field test of exploratory activity. **(b)** Time spent in the light and dark compartments (line plots) and total distance moved (box plot) in the light-dark box test of innate anxiety-like behavior. $^{+++}p < 0.001$, reflecting the significant main effect of compartment revealed by repeated-measures ANOVA ($F_{(1,18)} = 67.5$). All data are means ± SEM with individual values overlaid. Each data point represents one animal (experimental unit). The sample size for each group was $n = 10$. Source data are provided as a Source Data file.

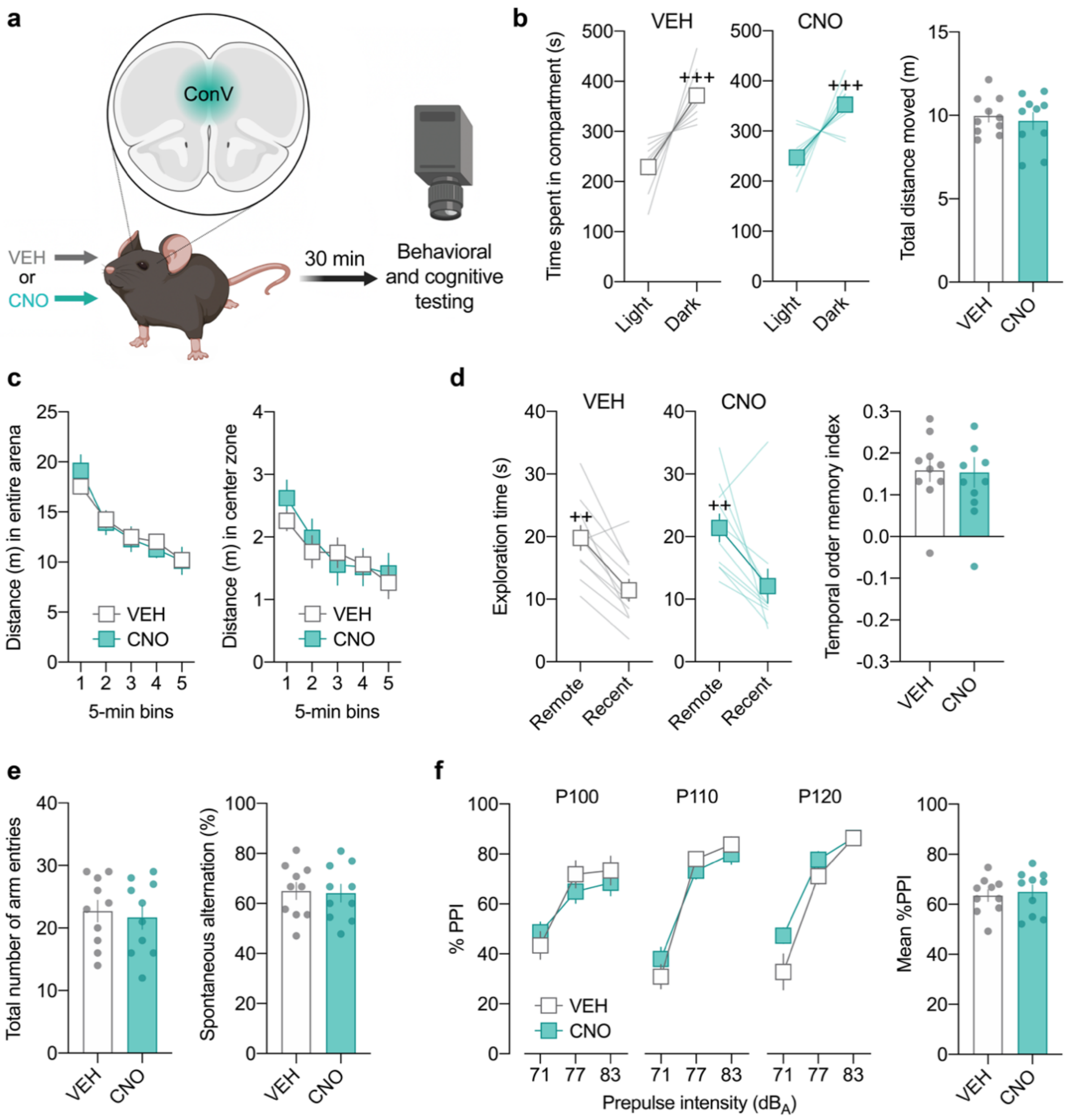

Figure S5. Clozapine-N-oxide treatment in ConV-expressing control mice does not alter behavior and cognition. (a) Male mice expressing a control AAV9-hGFAP-EGFP (ConV) in the prefrontal cortex were orally treated with vehicle (VEH) or CNO (1 mg/kg) and were then subjected to behavioral and cognitive testing 30 min after treatment (Created in BioRender. Notter, T. (2026) https://BioRender.com/djsd5lb). **(b)** Time spent in the light and dark compartments (line plots) and total distances moved (box plot) in the light-dark box test of innate anxiety-like behavior. $^{+++}p < 0.001$, reflecting the significant main effect of compartment revealed by repeated-measure ANOVA ($F_{(1,18)}$ = 37.9). **(c)** Distance moved in the entire arena and center zone during the open field test of exploratory activity. **(d)** Absolute exploration times of the temporally remote and recent objects (line plots) and temporal order memory index (bar plot) in the temporal order memory test for objects. $^{++}p < 0.01$, reflecting the significant main effect of object in VEH-treated ($F_{(1,9)}$ = 10.3) and CNO-treated ($F_{(1,9)}$ = 11.7) mice revealed by repeated-measure ANOVA. **(e)** Total number of arm entries and percent spontaneous alternation in the Y-maze test of working memory. **(f)** Prepulse inhibition (PPI) test of pre-attentive filtering. The line plots show % PPI as a function of prepulse intensity (71, 77, and 83 dB_A) for each of the three pulse conditions (P100, P110 and P120, which correspond to pulse intensities of 100, 110, and 120 dB_A). The bar plot depicts the mean % PPI across all prepulse and pulse intensities. All data are means ± SEM with individual values overlaid. Each data point represents one animal (experimental unit). The sample size for each group was n = 10. Source data are provided as a Source Data file.

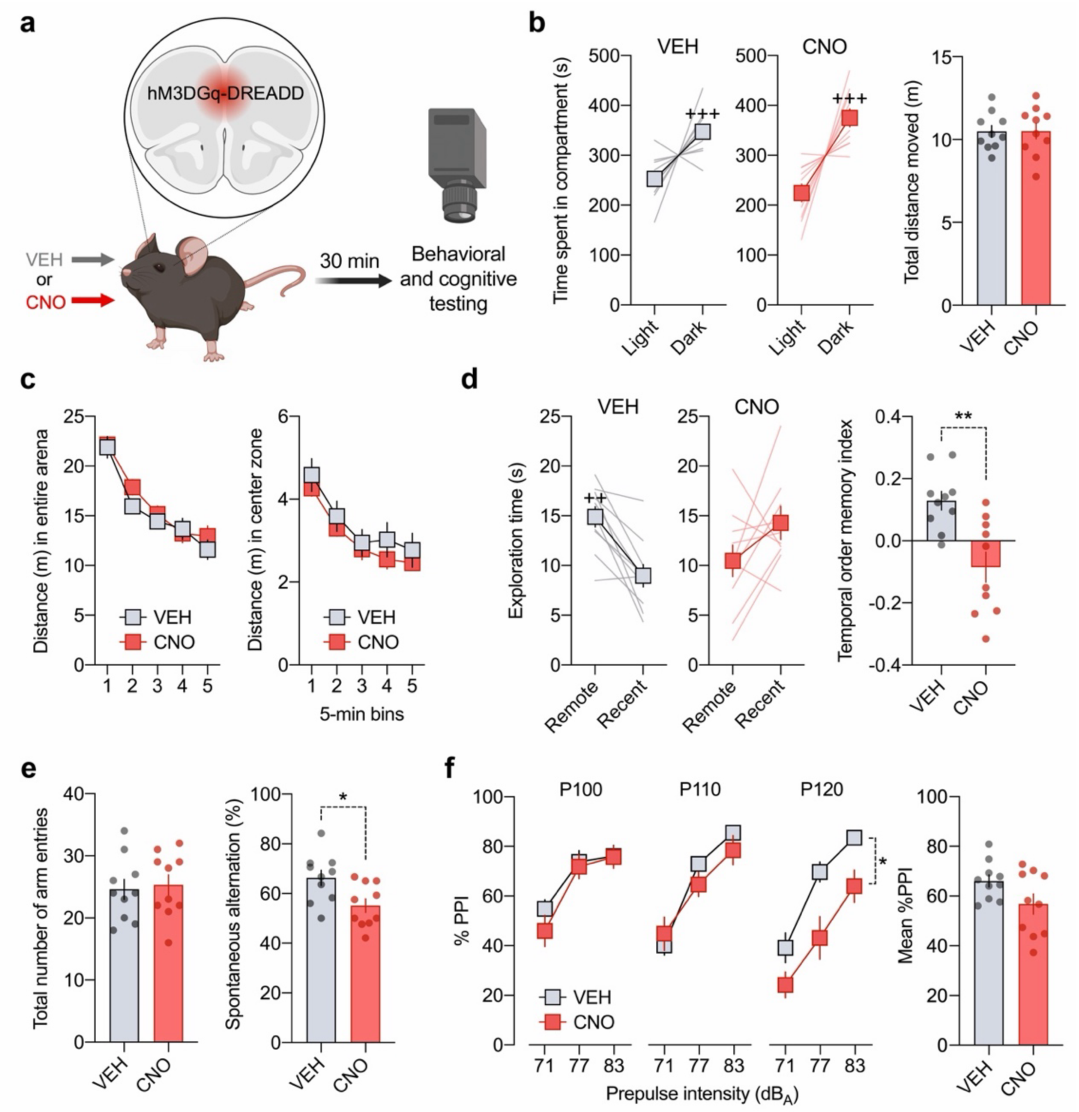

Figure S6. Chemogenetic activation of prefrontal astrocytes impairs cognition in female mice. (a) Female mice expressing hM3DGq in the prefrontal cortex were treated with vehicle (VEH) or clozapine-N-oxide (CNO, 1 mg/kg) and were subjected to behavioral and cognitive testing 30 min after treatment (Created in BioRender. Notter, T. (2026) https://BioRender.com/djsd5lb). **(b)** Time spent in the light and dark compartments (line plots) and total distances moved (box plot) in the light-dark box test of innate anxiety-like behavior. $^{+++}p < 0.001$, reflecting the significant main effect of compartment revealed by repeated-measure ANOVA ($F_{(1,18)} = 28.7$). **(c)** Distance moved in the entire arena and center zone during the open field test of exploratory activity. **(d)** Absolute exploration times of the temporally remote and recent objects (line plots) and temporal order memory index (bar plot) in the temporal order memory test for objects. $^{++}p < 0.01$, reflecting the significant main effect of object in VEH-treated mice revealed by repeated-measure ANOVA ($F_{(1,9)} = 15.4$); $^{**}p < 0.01$, $t_{(18)} = 3.75$ (two-tailed). **(e)** Total number of arm entries and percent spontaneous alternation in the Y-maze test of working memory. $^{*}p < 0.05$, $t_{(18)} = 2.70$ (two-tailed). **(f)** Prepulse inhibition (PPI) test of pre-attentive filtering. The line plots show % PPI as a function of prepulse intensity (71, 77, and 83 dB_A) for each of the three pulse conditions (P100, P110, and P120, which correspond to pulse intensities of 100, 110 and 120 dB_A). The bar plot depicts the mean % PPI across all prepulse and pulse intensities. $^{*}p < 0.05$, reflecting the significant main effect of treatment in the P120 condition ($F_{(1,18)} = 7.82$), based on repeated-measures ANOVA conducted for the P120 pulse condition following the presence of a significant main effect of pulse ($F_{(2,36)} = 6.84$, $p < 0.01$) and a treatment × pulse interaction ($F_{(2,36)} = 3.63$, $p < 0.05$) in the preceding full-factorial 2 × 3 × 3 (treatment × pulse × prepulse) repeated-measures ANOVA. All data are means ± SEM. Each data point represents one animal (experimental unit). The sample size for each group was $n = 10$. Source data are provided as a Source Data file.

Test	Dependent measure	Statistical outcomes
Temporal order memory test	Temporal order memory index	Main effect of treatment: $F_{(1,36)} = 29.96$, $p < 0.001$<br>Main effect of sex: $F_{(1,36)} = 0.15$, $p = 0.697$<br>Treatment × sex interaction: $F_{(1,36)} = 0.05$, $p = 0.825$
	Absolute exploration times (s) of objects	Main effect of treatment: $F_{(1,36)} = 3.65$, $p = 0.064$<br>Main effect of sex: $F_{(1,36)} = 1.77$, $p = 0.192$<br>Treatment × sex interaction: $F_{(1,36)} = 4.28$, $p = 0.046$<br>Main effect of object: $F_{(1,36)} = 4.18$, $p = 0.048$<br>Object × treatment interaction: $F_{(1,36)} = 26.62$, $p < 0.001$<br>Object × sex interaction: $F_{(1,36)} = 0.92$, $p = 0.343$<br>Object ×treatment × sex interaction: $F_{(1,36)} = 1.23$, $p = 0.276$
Y-maze working memory test	Percent alternation	Main effect of treatment: $F_{(1,36)} = 19.86$, $p < 0.001$<br>Main effect of sex: $F_{(1,36)} = 0.09$, $p = 0.768$<br>Treatment × sex interaction: $F_{(1,36)} = 0.44$, $p = 0.509$
	Total number of arm entries	Main effect of treatment: $F_{(1,36)} = 0.03$, $p = 0.856$<br>Main effect of sex: $F_{(1,36)} = 18.36$, $p < 0.001$<br>Treatment × sex interaction: $F_{(1,36)} = 0.48$, $p = 0.491$
Prepulse inhibition (PPI) test	Percent PPI	Main effect of treatment: $F_{(1,36)} = 12.82$, $p = 0.001$<br>Main effect of sex: $F_{(1,36)} = 0.26$, $p = 0.612$<br>Treatment × sex interaction: $F_{(1,36)} = 0.57$, $p = 0.457$<br>Main effect of pulse: $F_{(2,72)} = 8.74$, $p < 0.001$<br>Pulse × treatment: $F_{(2,72)} = 2.48$, $p = 0.09$<br>Pulse × sex: $F_{(2,72)} = 5.42$, $p = 0.006$<br>Pulse × treatment × sex: $F_{(2,72)} = 4.07$, $p = 0.021$
	Startle reactivity to pulse alone trials	Main effect of treatment: $F_{(1,36)} = 0.02$, $p = 0.888$<br>Main effect of sex: $F_{(1,36)} = 12.55$, $p = 0.001$<br>Treatment × sex interaction: $F_{(1,36)} = 4.17$, $p = 0.049$<br>Main effect of pulse: $F_{(2,72)} = 75.41$, $p < 0.001$<br>Pulse × treatment: $F_{(2,72)} = 0.26$, $p = 0.77$<br>Pulse × sex: $F_{(2,72)} = 4.91$, $p = 0.010$<br>Pulse × treatment × sex: $F_{(2,72)} = 1.10$, $p = 0.338$
Open field test	Distance moved (m) in entire arena	Main effect of treatment: $F_{(1,36)} = 0.45$, $p = 0.509$<br>Main effect of sex: $F_{(1,36)} = 23.81$, $p < 0.001$<br>Treatment × sex interaction: $F_{(1,36)} = 0.22$, $p = 0.639$<br>Main effect of bin: $F_{(4,144)} = 136.95$, $p < 0.001$<br>Bin × treatment: $F_{(4,144)} = 1.80$, $p = 0.132$<br>Bins × sex: $F_{(4,144)} = 1.33$, $p = 0.261$<br>Bins × treatment × sex: $F_{(4,144)} = 0.91$, $p = 0.462$
	Distance moved (m) in center zone	Main effect of treatment: $F_{(1,36)} = 2.58$, $p = 0.117$<br>Main effect of sex: $F_{(1,36)} = 57.64$, $p < 0.001$<br>Treatment × sex interaction: $F_{(1,36)} = 0.08$, $p = 0.774$<br>Main effect of bins: $F_{(4,144)} = 97.92$, $p < 0.001$<br>Bins × treatment: $F_{(4,144)} = 0.47$, $p = 0.759$<br>Bins × sex: $F_{(4,144)} = 3.18$, $p = 0.015$<br>Bins × treatment × sex: $F_{(4,144)} = 0.11$, $p = 0.98$
Light-dark box test	Time spent (s) in compartment	Main effect of treatment: $F_{(1,36)} = 1.85$, $p = 0.183$<br>Main effect of sex: $F_{(1,36)} = 0.46$, $p = 0.501$<br>Treatment × sex interaction: $F_{(1,36)} = 0.46$, $p = 0.501$<br>Main effect of compartment: $F_{(1,36)} = 92.68$, $p < 0.001$<br>Compartment × treatment: $F_{(1,36)} = 2.79$, $p = 0.104$<br>Compartment × sex: $F_{(1,36)} = 4.72$, $p = 0.036$<br>Compartment × treatment × sex: $F_{(1,36)} = 0.001$, $p = 0.973$
	Total distance moved (m)	Main effect of treatment: $F_{(1,36)} = 0.41$, $p = 0.527$<br>Main effect of sex: $F_{(1,36)} = 5.55$, $p = 0.024$<br>Treatment × sex interaction: $F_{(1,36)} = 0.15$, $p = 0.704$

Supplementary Table S1: Summary of the statistical outcomes of full-factorial ANOVAs, in which both treatment and sex were included as independent factors to analyze the behavioral and cognitive effects of hM3DGq-induced activation of prefrontal astrocytes. The table summarizes the dependent measures for each test and reports the relevant main effects and interactions.

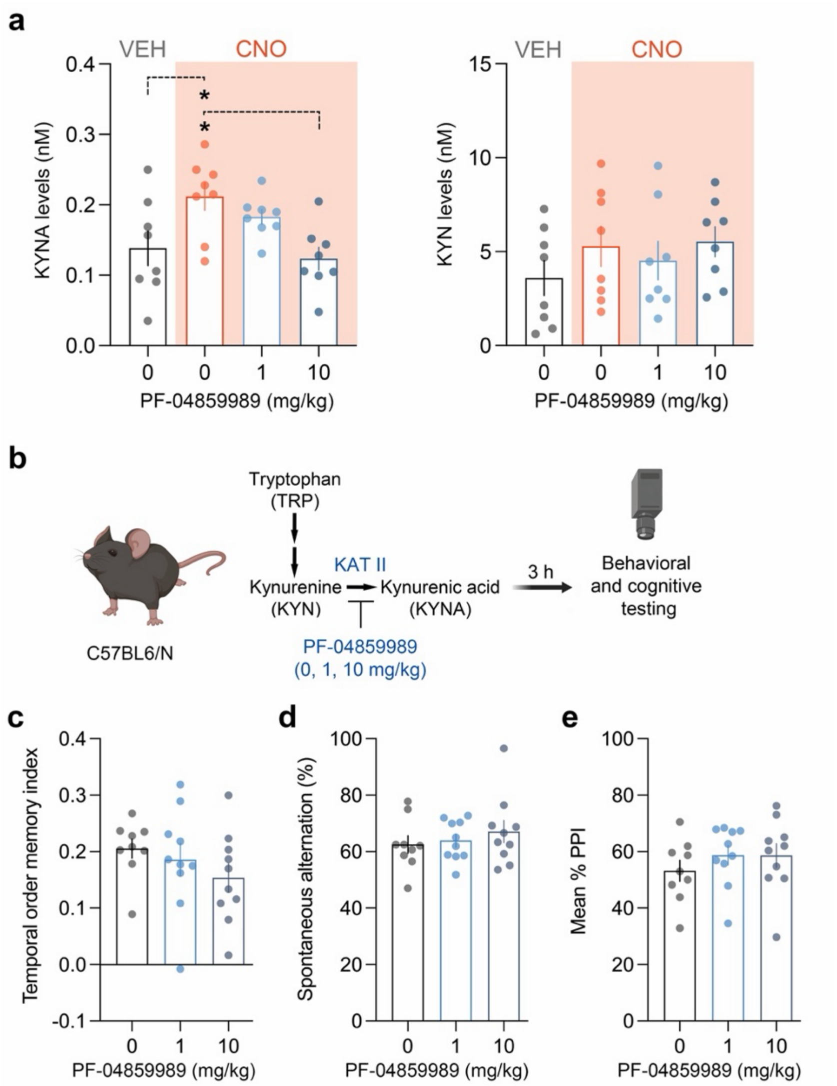

Figure S7. Blocking kynurenic acid (KYNA) synthesis prevents KYNA elevation in response to prefrontal astrocyte stimulation but has no pro-cognitive effect in C57BL6/N mice. (a) hM3DGq-expressing mice were pretreated with the kynurenine aminotransferase II (KAT II) inhibitor PF-04859989 (0, 1, or 10 mg/kg, i.p.) 2.5 hours before receiving clozapine-N-oxide (CNO, 1 mg/kg) or vehicle (VEH). 30 min after CNO or VEH administration brains were collected for metabolite measurements. *$p < 0.05$, based on Tukey's post-hoc test following ANOVA ($F_{(3,28)} = 1.866$, $p < 0.01$). Each data point represents the sample of one mouse (experimental unit). The sample size for each group was $n = 8$. **(b)** Male C57BL6/N mice received 0, 1 or 10 mg/kg PF-04859989 and were subjected to behavioral and cognitive testing 3 hours (h) after treatment (Created in BioRender. Notter, T. (2026) https://BioRender.com/lshtqyt). **(c)** Temporal order memory index in the temporal order memory test for objects. **(d)** Percent spontaneous alternation in the Y-maze test of working memory. **(e)** Prepulse inhibition (PPI) test of pre-attentive filtering. All data are means ± SEM, with individual data points overlaid. **(c-e)** Each data point represents one animal (experimental unit). The sample size for each group was $n = 10$ and $n = 9$ (0 mg/kg). Source data are provided as a Source Data file. Schematic was created with BioRender.com.

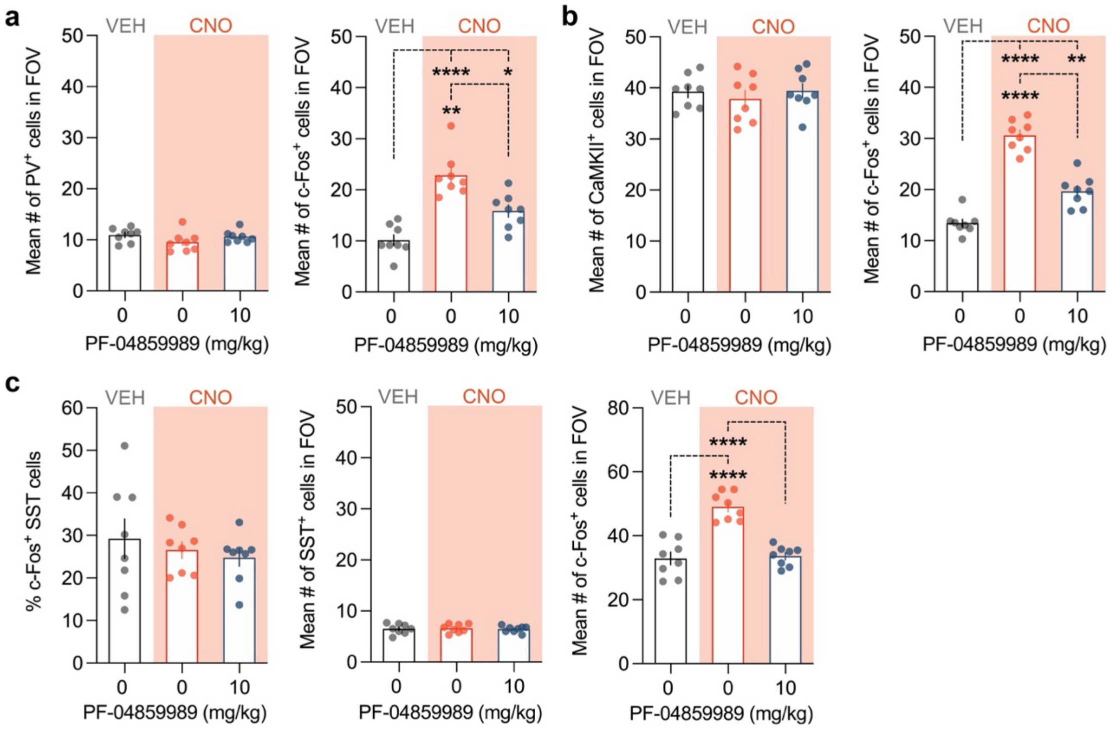

Figure S8. Blocking KYNA synthesis restores astrocyte-induced changes in prefrontal neuronal activity pattern. Cell-type-specific c-Fos mapping in the prefrontal cortex (PFC) of vehicle (VEH)-treated hM3DGq mice receiving vehicle (0 mg/kg PF-04859989) only, and in hM3DGq mice receiving clozapine-N-oxide (CNO, 1 mg/kg) in combination with 0 or 10 mg/kg PF-04859989. **(a)** c-Fos expression in parvalbumin (PV)+ interneurons. The bar plots depict the mean number (#) of PV positive cells and the mean # of c-Fos positive cells in the field of view (FOV). **p* < 0.05, ***p* < 0.01 and *****p* < 0.0001, based on Tukey's post-hoc test following one-way ANOVA ($F_{(2,21)}$ = 24.72, p < 0.0001). **(b)** c-Fos expression in calmodulin-dependent protein kinase II (CaMKII)+ pyramidal cells. The bar plots depict the mean # of CaMKII positive cells and the mean # c-Fos positive cells in the FOV. ***p* < 0.01 and *****p* < 0.0001, based on Tukey's post-hoc test following one-way ANOVA ($F_{(2,21)}$ = 79.59, p < 0.0001). **(c)** c-Fos expression in somatostatin (SST)+ interneurons. The bar plots depict the % of c-Fos positive SST interneurons, the mean # of SST+ cells, and the mean # of c-Fos positive cells in the FOV. *****p* < 0.0001, based on Tukey's post-hoc test following one-way ANOVA ($F_{(2,21)}$ = 32.96, p < 0.0001). All data are means ± SEM with individual values overlaid. Each data point represents one animal (experimental unit). The sample size for each group was n = 8. Source data are provided as a Source Data file.

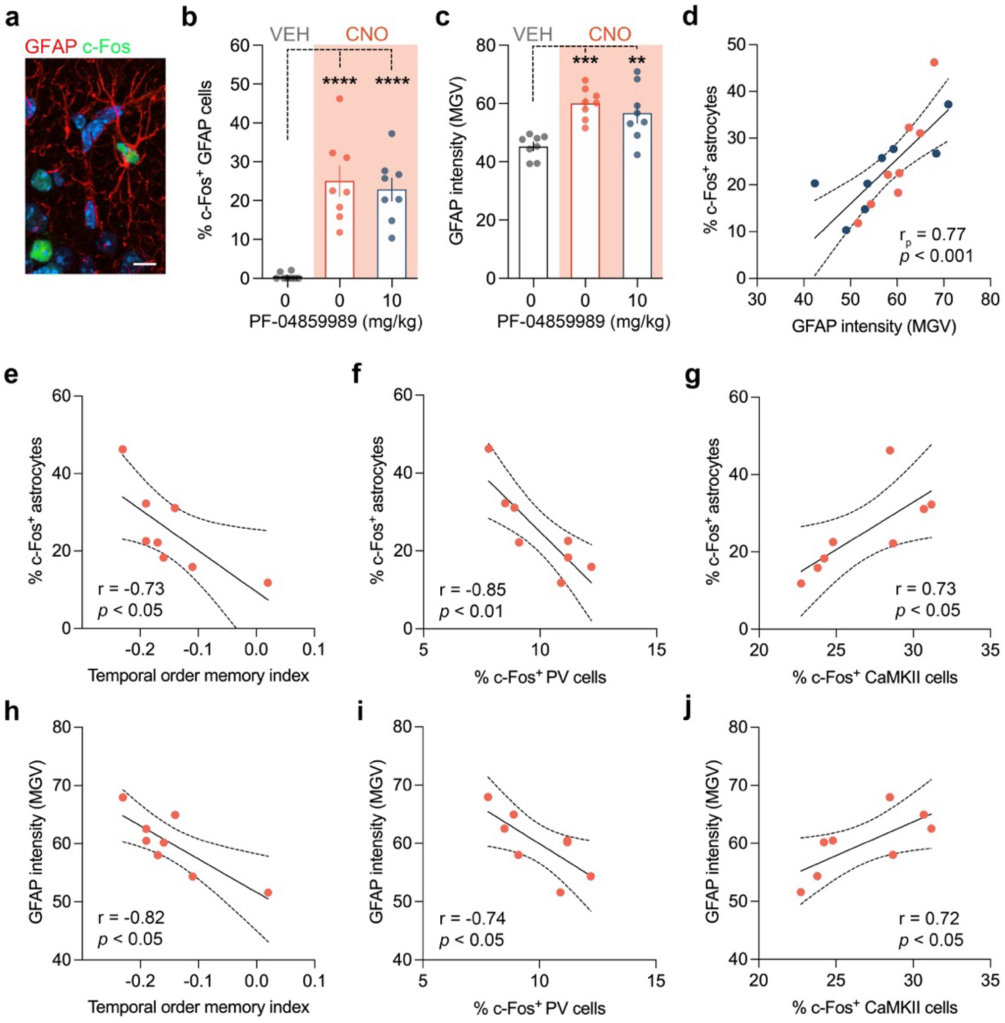

Figure S9. Correlations between the magnitude of DREADD-induced activation of prefrontal astrocytes and magnitude of the behavioral and cellular outcomes. Astrocyte-specific c-Fos expression and glial fibrillary acidic protein (GFAP) intensity were measured in the prefrontal cortex (PFC) of vehicle (VEH)-treated hM3DGq mice receiving vehicle (0 mg/kg PF-04859989) only, and in hM3DGq mice receiving clozapine-N-oxide (CNO, 1 mg/kg) in combination with 0 or 10 mg/kg PF-04859989. Measures of astrocyte activation were then correlated with behavioral and cellular outcomes following hM3DGq activation. **(a)** Representative image of a c-Fos-positive (green) astrocyte (immunoreactive for GFAP, red) following hM3DGq-induced activation. Cell nuclei were visualized with DAPI. Scale bar = 10 µm. **(b)** % of c-Fos-positive GFAP-positive astrocytes. ****$p < 0.0001$, based on Tukey's post-hoc test following one-way ANOVA ($F_{(2,21)} = 23.06$, $p < 0.0001$). **(c)** Mean intensity of GFAP (mean grey value (MGV)). **$p < 0.01$, ***$p < 0.001$, based on Tukey's post-hoc test following one-way ANOVA ($F_{(2,21)} = 10.62$, $p < 0.001$). All data are means ± SEM with individual values overlaid. **(d)** Partial correlation between % c-Fos-positive astrocytes and GFAP intensity following hM3DGq stimulation, controlling for the effects of PF-04859989 treatment. Mice receiving 0 mg/kg PF-04859989 are highlighted in orange and mice receiving 10 mg/kg PF-04859989 are highlighted in blue. **(e)** Pearson's product moment correlations between % c-Fos-positive astrocytes and temporal order memory index. **(f)** Pearson's product moment correlations between % c-Fos-positive astrocytes and % c-Fos-positive PV+ cells. **(g)** Pearson's product moment correlations between % c-Fos-positive astrocytes and % c-Fos-positive CaMKII pyramidal cells. **(h)** Pearson's product moment correlations between GFAP intensity and temporal order memory index. **(i)** Pearson's product moment correlations between GFAP intensity and % c-Fos-positive PV+ cells. **(j)** Pearson's product moment correlations between GFAP intensity and % c-Fos-positive CaMKII pyramidal cells. Source data are provided as a Source Data file.

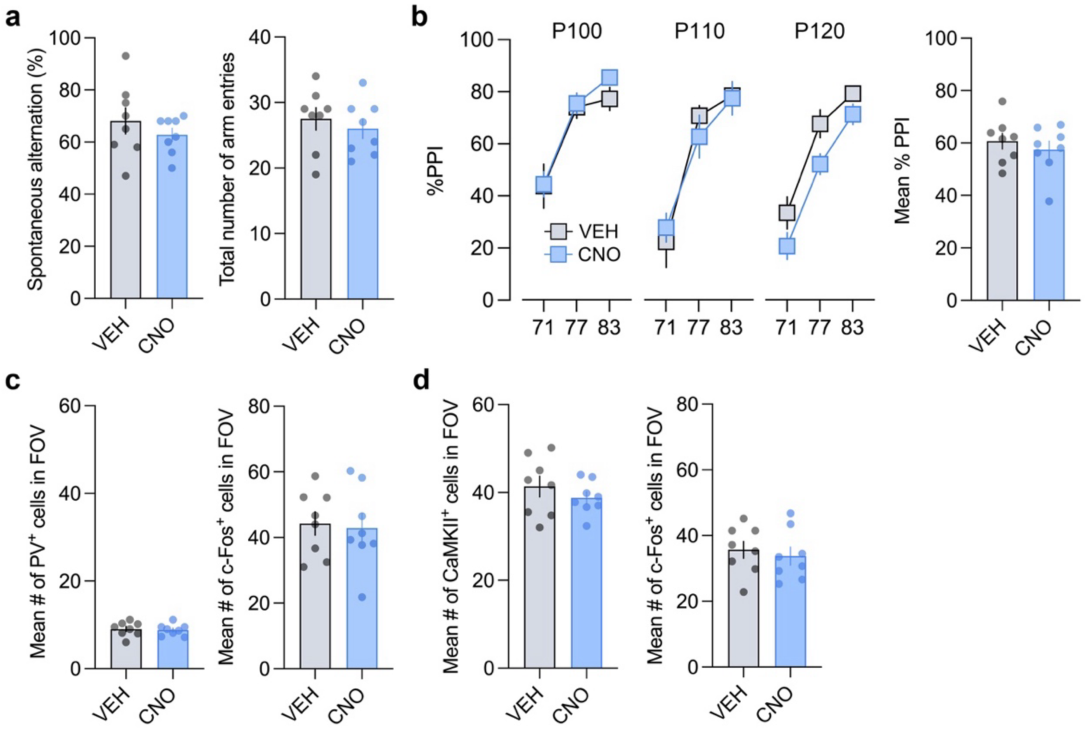

Figure S10. KAT II knockdown prevents astrocyte-mediated cognitive and neuronal deficits. Male mice expressing $KATII^{KD}$-hM3DGq in prefrontal astrocytes were treated with vehicle (VEH) or clozapine-N-oxide (CNO, 1 mg/kg) 30 min prior to cognitive testing. After the last test, animals were left undisturbed for one week, after which they were again treated with either VEH or CNO. 2 hours after treatment, brains were collected for subsequent c-Fos mapping. **(a)** Percent spontaneous alternation and total number of arm entries in the Y-maze test of working memory. **(b)** Prepulse inhibition (PPI) test of pre-attentive filtering. The line plots show % PPI as a function of prepulse intensity (71, 77 and 83 dB_A) for each of the three pulse conditions (P100, P110 and P120, which correspond to pulse intensities of 100, 110 and 120 dB_A). The bar plot depicts the mean % PPI across all prepulse and pulse intensities. **(c)** c-Fos expression in parvalbumin (PV)+ interneurons. The bar plots depict the mean # of PV+ cells and the mean # of c-Fos positive cells in the FOV. **(d)** c-Fos expression in calmodulin-dependent protein kinase II (CaMKII)+ pyramidal cells. The bar plots depict the mean # of CaMKII+ cells and the mean # of c-Fos positive cells in the FOV. All data are means ± SEM with individual values overlaid. Each data point represents one animal (experimental unit). The sample size for each group was *n* = 8. Source data are provided as a Source Data file.

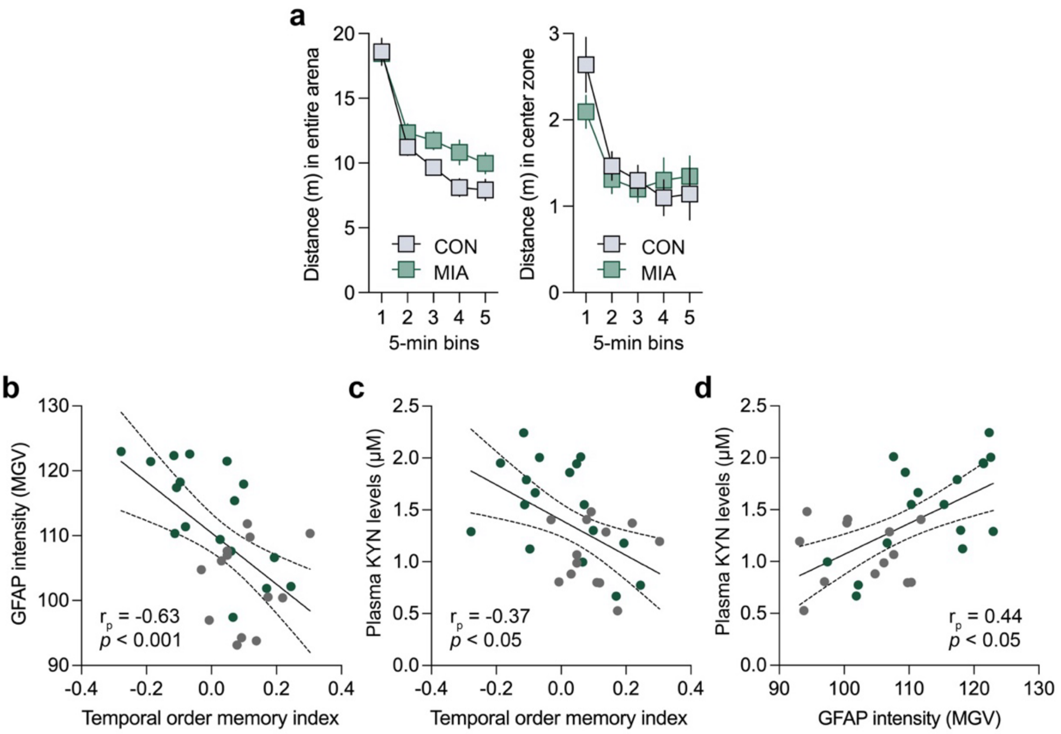

Figure S11. Open field test and correlations between astrocytic, cognitive and metabolic measures in the maternal immune activation model. Pregnant mice were exposed to 0.9% NaCl (CON) treatment or maternal immune activation (MIA) induced by poly(I:C) administration on gestation day (GD) 12. The resulting male offspring underwent testing once they reached adulthood. **(a)** The line plots show the distance moved as a function of 5-min bins in the entire arena (left) and center zone (right) during the open field test. **(b)** Partial correlations between prefrontal GFAP intensity and temporal order memory index across all offspring, controlling for prenatal treatment effects. MIA offspring are highlighted with green and CON offspring with grey dots. **(c)** Partial correlations between plasma KYN levels and temporal order memory index across all offspring, controlling for prenatal treatment effects. MIA offspring are highlighted with green and CON offspring with grey dots. **(d)** Partial correlations between plasma KYN levels and prefrontal GFAP intensity across all offspring, controlling for prenatal treatment effects. MIA offspring are highlighted with green and CON offspring with grey dots. All data are means ± SEM. Each data point represents one animal (experimental unit). The sample size for each group was n = 13 (CON) and n = 17 (MIA). Source data are provided as a Source Data file.

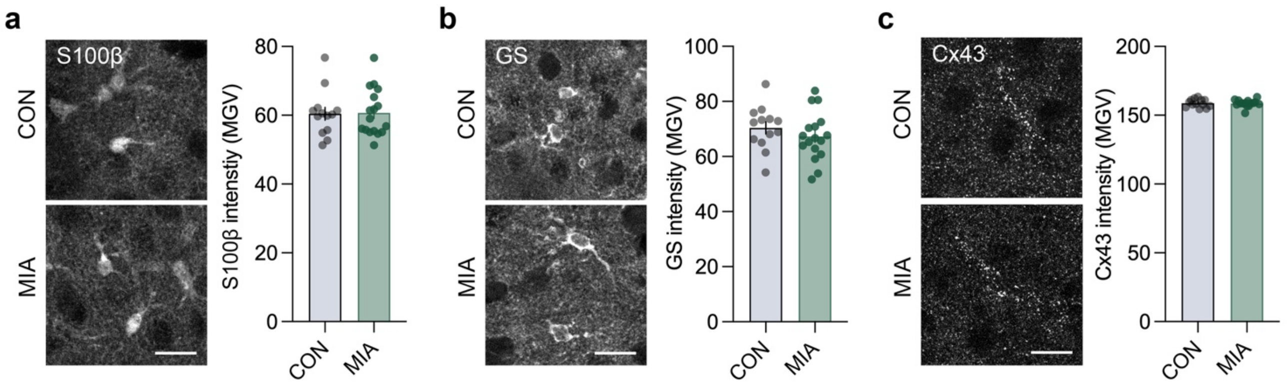

Figure S12. Immunohistochemical analyses of astrocyte markers in the maternal immune activation model. Immunohistochemical analyses of astrocyte markers were conducted in the prefrontal cortex (PFC, spanning anterior cingulate, prelimbic, and infralimbic cortices) once the offspring reached adulthood. **(a)** Representative stains against S100β corresponding mean intensity (mean grey value, MGV). **(b)** Representative stains against glutamine synthetase (GS) and corresponding mean intensity (MGV). **(c)** Representative stains against connexin 43 (Cx43) and corresponding mean intensity (MGV). All data are means ± SEM with individual values overlaid. Each data point represents one animal (experimental unit). The sample size for each group was *n* = 13 (CON) and *n* = 17 (MIA). Scale bars = 20 μm. Source data are provided as a Source Data file.
