# Supplementary material for: Astroglial disinhibition of cortical circuits disrupts cognition via kynurenic acid in mice
Source: Nat Commun. 2026 May 8;17:6210. doi: 10.1038/s41467-026-72640-0 (PMC13369966; doi:10.1038/s41467-026-72640-0)
Supplement: Supplementary file 2 — Reporting Summary [file 41467_2026_72640_MOESM2_ESM.pdf]

nature portfolio

Corresponding author(s): Tina Notter

Last updated by author(s): Mar 11, 2026

Reporting Summary

Nature Portfolio wishes to improve the reproducibility of the work that we publish. This form provides structure for consistency and transparency in reporting. For further information on Nature Portfolio policies, see our Editorial Policies and the Editorial Policy Checklist.

Statistics

For all statistical analyses, confirm that the following items are present in the figure legend, table legend, main text, or Methods section.

n/a	Confirmed	
☐	☒	The exact sample size (*n*) for each experimental group/condition, given as a discrete number and unit of measurement
☐	☒	A statement on whether measurements were taken from distinct samples or whether the same sample was measured repeatedly
☐	☒	The statistical test(s) used AND whether they are one- or two-sided<br>*Only common tests should be described solely by name; describe more complex techniques in the Methods section.*
☒	☐	A description of all covariates tested
☐	☒	A description of any assumptions or corrections, such as tests of normality and adjustment for multiple comparisons
☐	☒	A full description of the statistical parameters including central tendency (e.g. means) or other basic estimates (e.g. regression coefficient) AND variation (e.g. standard deviation) or associated estimates of uncertainty (e.g. confidence intervals)
☐	☒	For null hypothesis testing, the test statistic (e.g. *F*, *t*, *r*) with confidence intervals, effect sizes, degrees of freedom and *P* value noted<br>*Give P values as exact values whenever suitable.*
☒	☐	For Bayesian analysis, information on the choice of priors and Markov chain Monte Carlo settings
☒	☐	For hierarchical and complex designs, identification of the appropriate level for tests and full reporting of outcomes
☐	☒	Estimates of effect sizes (e.g. Cohen's *d*, Pearson's *r*), indicating how they were calculated

Our web collection on statistics for biologists contains articles on many of the points above.

Software and code

Policy information about availability of computer code

Data collection	- Confocal microscopy and image analysis: Zeiss LSM800 with Airyscan, ZEN 2.6 blue edition software (Zeiss, Switzerland), ImageJ software (ImageJ 1.53k, NIH, USA)<br>- Two-photon microscopy and image analysis: customized version of "ScanImage" (r3.8.1; Janelia Research Campus), ImageJ software (ImageJ 1.53k, NIH, USA)<br>- Behavioral testing: Multi conditioning system (version 0.7.34, TSE Systems, Germany); EthoVision tracking system (version 14.0, Noldus Information Technology, The Netherlands); San Diego Instruments (SR_lab software 2014-2015, USA) for prepulse inhibition of the acoustic startle response<br>- 2024 Microsoft Excel for Mac (version 16.85)<br>- TSQ Quantiva triple quadrupole mass spectrometer (ThermoFisher Scientific, United States) coupled to an ACQUITY UPLC M-Class (Waters, United States).<br>- C18 reversed-phase HPLC column (100 mm × 4 mm; Dr. Maisch GmbH, Ammerbuch, Germany)<br>- Jasco FP-2020 Plus spectrofluorometer (Jasco Inc., Tokyo, Japan)<br>- Nanodrop (DeNovix DS-11+ spectrophotometer, Labgene Scientific SA, Switzerland)<br>- Qubit 4 Fluorometer A.0 (MAN0017210, Invitrogen, Switzerland)<br>- TaqMan qRT-PCR instrument (CFX384 real-time system, Bio-Rad Laboratories)<br>- TissueLyserII (21920, Qiagen, Germany)<br>- Lab™ Upgrade for ChemiDoc™ XRS+ System (1708299, Bio-Rad, USA)<br>- Mini-PROTEAN Tetra Vertical Electrophoresis Cell (1658004, Bio-Rad, USA)<br>- The Trans-Blot® TurboTM Transfer System (1704150, BioRad, USA)
Data analysis	- SPSS Statistics (version 28.0, IBM, Armonk, NY, USA)<br>- Prism (version 10.5; GraphPad Software, La Jolla, CA, USA)<br>- 2024 Microsoft Excel for Mac (version 16.85)<br>- ImageJ software (ImageJ 1.53k, NIH, USA)

For manuscripts utilizing custom algorithms or software that are central to the research but not yet described in published literature, software must be made available to editors and reviewers. We strongly encourage code deposition in a community repository (e.g. GitHub). See the Nature Portfolio guidelines for submitting code & software for further information.

Data

Policy information about availability of data

All manuscripts must include a data availability statement. This statement should provide the following information, where applicable:

- Accession codes, unique identifiers, or web links for publicly available datasets
- A description of any restrictions on data availability
- For clinical datasets or third party data, please ensure that the statement adheres to our policy

All data are included in the manuscript or the supplementary information. Source data are provided with this paper.

Research involving human participants, their data, or biological material

Policy information about studies with human participants or human data. See also policy information about sex, gender (identity/presentation), and sexual orientation and race, ethnicity and racism.

Reporting on sex and gender	This study did not involve human participants, their data or biological material derived from humans.
Reporting on race, ethnicity, or other socially relevant groupings	This study did not involve human participants, their data or biological material derived from humans.
Population characteristics	This study did not involve human participants, their data or biological material derived from humans.
Recruitment	This study did not involve human participants, their data or biological material derived from humans.
Ethics oversight	This study did not involve human participants, their data or biological material derived from humans.

Note that full information on the approval of the study protocol must also be provided in the manuscript.

Field-specific reporting

Please select the one below that is the best fit for your research. If you are not sure, read the appropriate sections before making your selection.

☒ Life sciences ☐ Behavioural & social sciences ☐ Ecological, evolutionary & environmental sciences

For a reference copy of the document with all sections, see nature.com/documents/nr-reporting-summary-flat.pdf

Life sciences study design

All studies must disclose on these points even when the disclosure is negative.

Sample size	No statistical tests were used to pre-determine sample sizes. The sample sizes were chosen based on our extensive experiences in conducting behavioral and cognitive tests as well as postmortem analyses (Notter et al., 2018, Schalbetter et al., 2021; Schalbetter et al., 2022). Sample size for each experiment was indicated in each figure legend and in supplementary table 1 and supplementary table 2.
Data exclusions	No data was excluded.
Replication	All experiments were independently and successfully replicated, except for the two-photon imaging experiments involving KATIIKD-hM3DGq or PV interneuron activity and the pharmacological rescue experiments in the MIA model, which were performed once.
Randomization	For all experiments (except for two-photon imaging experiment) animals were randomly assigned to different treatment groups using the online randomization tool (http://www.randomizer.org). As an example, to assign animals to two treatments (VEH or CNO) the following randomization was conducted: Prior to treatment allocation animals were numbered from 1-20. Using the randomization tool, 2 sets of 10 unique numbers per set with numbers ranging from 1-20 were created. Animals allocated to the first set were assigned to VEH treatment and animals allocated to set 2 were assigned to CNO treatment. For the MIA model, pregnant dam were randomly assigned to control or poly(I:C) treatment.
Blinding	All behavioral, cognitive, immunohistochemical and molecular data were acquired and analyzed in a blind manner, in which the treatment conditions were blinded in the form of numerical codes. Likewise, all samples collected for HPLC-FLD and LC-NSI-MS/MS analysis were randomly labeled by an experimenter before the measurements and analyses were conducted, with the samples being unblinded once all data were collected and analyzed.

Reporting for specific materials, systems and methods

We require information from authors about some types of materials, experimental systems and methods used in many studies. Here, indicate whether each material, system or method listed is relevant to your study. If you are not sure if a list item applies to your research, read the appropriate section before selecting a response.

Materials & experimental systems

n/a	Involved in the study	
☐	☒	Antibodies
☒	☐	Eukaryotic cell lines
☒	☐	Palaeontology and archaeology
☐	☒	Animals and other organisms
☒	☐	Clinical data
☒	☐	Dual use research of concern
☒	☐	Plants

Methods

n/a	Involved in the study	
☒	☐	ChIP-seq
☒	☐	Flow cytometry
☒	☐	MRI-based neuroimaging

Antibodies

Antibodies used	- GFAP, chicken, polyclonal, ab4674, Abcam, UK, dilution 1:2000<br>- s100beta, rabbit monoclonal (EP1576Y), ab52642, Abcam, Switzerland, dilution 1:1000<br>- GS, mouse monoclonal, 610518, BD Transduction Laboratories, dilution 1:1000<br>- Cx43, rabbit polyclonal, 3512, Cell Signaling, USA, dilution 1:1000<br>- mCherry, rat monoclonal (16D7), M11217, Invitrogen, Switzerland, dilution 1:1000<br>- Iba1, rabbit polyclonal, 019-19741, Wako Chemicals, USA, dilution 1:2000<br>- NeuN, rabbit monoclonal (EPR12763), ab177487, Abcam, Switzerland, Dilution 1:500<br>- HA-tag, goat, polyclonal, A190-138A, Fortis Life Sciences, USA, dilution 1:2000<br>- c-Fos, rabbit, monoclonal (9F6), 2250, Cell Signaling, USA, dilution 1:1000<br>- PV, guinea pig, polyclonal, 195 004, Synaptic Systems, Germany, dilution 1:1000<br>- CaMKII alpha, mouse, monoclonal (6G9), GeneTex, USA, dilution 1:500<br>- SST, mouse, monoclonal (C11), AA 27-116, Antibodies online, USA, dilution 1:500
Validation	All antibodies were commercially available and validated for the species and application by the company, as well as other researchers:<br>- GFAP: https://www.abcam.com/en-us/products/primary-antibodies/gfap-antibody-astrocyte-marker-ab4674<br>- s100beta: https://www.abcam.com/en-ch/products/primary-antibodies/s100-beta-antibody-ep1576y-astrocyte-marker-ab52642<br>- GS: https://www.bdbiosciences.com/en-ch/products/reagents/microscopy-imaging-reagents/immunofluorescence-reagents/purified-mouse-anti-glutamine-synthetase.610518<br>- Cx43: https://www.cellsignal.com/products/primary-antibodies/connexin-43-antibody/3512<br>- mCherry: https://www.thermofisher.com/antibody/product/mCherry-Antibody-clone-16D7-Monoclonal/M11217<br>- Iba1: https://www.labome.com/product/Wako-Chemicals-USA/019-19741.html

	- NeuN: https://www.abcam.com/en-gb/products/primary-antibodies/neun-antibody-epr12763-neuronal-marker-ab177487<br>- HA-tag: https://www.fortislife.com/products/primary-antibodies/goat-anti-ha-tag-antibody/BETHYL-A190-138<br>- c-Fos: https://www.cellsignal.com/products/primary-antibodies/c-fos-9f6-rabbit-mab/2250<br>- PV: https://www.citeab.com/antibodies/509493-195-004-parvalbumin-polyclonal-antibody<br>- CaMKII: https://www.genetex.com/Product/Detail/CaMKII-alpha-antibody-6G9/GTX41976<br>- SST: https://www.antibodies-online.com/antibody/6994841/anti-Somatostatin+SST+AA+27-116

Animals and other research organisms

Policy information about studies involving animals; ARRIVE guidelines recommended for reporting animal research, and Sex and Gender in Research

Laboratory animals	All experiments were performed using adult (10-week-old) male or female C57BL/6N mice (Charles Rivers, Sulzfeld, Germany) or transgenic PVCre mice (PV-Cre(B6;129P2-Pvalbtm1(cre)Arbr/JAX:008069).
Wild animals	Not applicable.
Reporting on sex	The effect of chemogenetic activation of prefrontal astrocytes on behavioral and cognitive functions was tested in male and female mice.<br>All other experiments involved male mice only.
Field-collected samples	Not applicable.
Ethics oversight	All procedures conducted within this study were approved by the Cantonal Veterinarian's Office of Zurich, Switzerland (ZH214/2022, ZH028/2023, ZH210/2024, ZH213/2025).

Note that full information on the approval of the study protocol must also be provided in the manuscript.

Plants

Seed stocks	Not applicable.
Novel plant genotypes	Not applicable.
Authentication	Not applicable.
