# Supplementary material for: Astroglial disinhibition of cortical circuits disrupts cognition via kynurenic acid in mice
Source: Nat Commun. 2026 May 8;17:6210. doi: 10.1038/s41467-026-72640-0 (PMC13369966; doi:10.1038/s41467-026-72640-0)
Supplement: Supplementary file 3 — Transparent Peer Review file [file 41467_2026_72640_MOESM3_ESM.pdf]

nature portfolio

Peer Review File

Astroglial disinhibition of cortical circuits disrupts cognition via kynurenic acid in mice

Corresponding Author: Dr Tina Notter

This file contains all reviewer reports in order by version, followed by all author rebuttals in order by version.

Version 0:

Reviewer comments:

Reviewer #1

(Remarks to the Author)
The revised manuscript shows a substantial improvement over the previous version, both in overall clarity and in the strength of several newly added datasets. In particular, the expanded cellular analyses and the cell-type–targeted manipulation supporting an astrocytic source of KYNA significantly reinforce the main narrative. I congratulate the authors for the effort and for seriously engaging with many of the prior concerns.
However, a few key issues remain only partially addressed, mainly regarding mechanistic specificity, statistical rigor for sex-related claims, and interpretability of variability/expression–phenotype relationships. These points still limit the strength of some causal statements and should be addressed as follows.

1.- The rationale for using Gq-DREADDs is reasonable, but the manuscript still does not test whether the reported behavioral/circuit effects are specific to PLC–IP3/Ca^{2+} signaling versus other astrocytic signaling pathways (e.g., Gi/Gs/cAMP). The added MIA model supports broader relevance but does not resolve this mechanistic specificity question. At minimum, mechanistic language should be tempered.

2.- Claims that effects are “similar” across sexes are not supported by formal statistics (e.g., sex × treatment interaction or direct male–female comparisons). Please either provide these tests.

3.- The manuscript does not address whether phenotype magnitude relates to the extent of astrocyte DREADD expression/activation in the DREADD cohorts. The MIA GFAP correlation is interesting but not a direct substitute. A correlation between expression/coverage (or activation readouts) and behavioral/cellular outcomes would strengthen interpretation.

Reviewer #2

(Remarks to the Author)
The manuscript Astroglial disinhibition of cortical circuits disrupts cognition via kynurenic acid by Beilmann et al. demonstrates that increased synthesis of KYNA in astrocytes can inhibit PV interneurons in the prefrontal cortex and cause cognitive impairments. The work is performed using targeted genetic manipulations and in vivo calcium recordings, thus providing relevant experimental evidence. Moreover, the authors include a mouse model of neurodevelopmental disorders and show that this mechanism is activated in this model.

The authors have made significant additions compared with an earlier version of the paper and now provide a comprehensive study of astrocytic KYNA in the prefrontal cortex. The experiments are well performed and the manuscript is well written. The data are well presented and appropriate controls are used. The work is relevant in a complex field and provides an important piece of the puzzle of astrocyte–neuron interactions.

I have a few minor comments:
The authors imply that increased KYNA is causative in cognitive dysfunction and part of the pathophysiological process. When KAT is reduced, the effect of DREADD-induced activation on cognition and c-Fos expression in PV neurons is abolished. However, the knockdown of KAT is partial and shows substantial variability, as does the exploration time in the

cognitive test. If the authors still have available samples from the animals that underwent cognitive testing, the paper could be strengthened by showing a correlation between KYNA levels and cognitive deficits. In addition, in the MIA model, a correlation between KYNA, GFAP, and plasma KYN is shown (Fig. 5), but not in controls. If KYNA is causative, such a correlation should exist regardless of disease model, and the authors could consider merging control and MIA animals in the same graph.

The statistics for PPI are reported differently across experiments and are not very convincing. In female mice (Supplementary Fig. S6f), significance is observed at a specific stimulus intensity, whereas in male mice significance is reported for the merged dataset (Fig. 2). This should be clarified, and the actual behavioral effect should be discussed.

The amounts of KYN and KYNA are reported using different units in Fig. 1 and Supplementary Fig. S7 so the results can not be comapred. In the first experiment, DREADD activation of astrocytes reduces KYN levels, whereas in the second experiment it does not. This discrepancy should be addressed.
There is a vast literature on the kynurenine pathway, and the roles of individual metabolites are complex and difficult to disentangle. Moreover, the authors mention that KYNA inhibits NMDA receptors, but several other receptors may also be involved. This should be more clearly acknowledged in introduction and discussion.

Reviewer #3

(Remarks to the Author)
The authors have addressed my main comments. Although the manuscript has improved significantly, it would be important to discuss the key role of astrocytes metabolism on cognitive function. This will increase the translational impact of the study.

Version 1:

Reviewer comments:

Reviewer #1

(Remarks to the Author)
The authors have addressed the main concerns raised during review, and the manuscript is now acceptable in its revised form

Reviewer #2

(Remarks to the Author)
The authors have adressed all my concerns.

Open Access This Peer Review File is licensed under a Creative Commons Attribution 4.0 International License, which permits use, sharing, adaptation, distribution and reproduction in any medium or format, as long as you give appropriate credit to the original author(s) and the source, provide a link to the Creative Commons license, and indicate if changes were

made.
In cases where reviewers are anonymous, credit should be given to 'Anonymous Referee' and the source.
The images or other third party material in this Peer Review File are included in the article’s Creative Commons license, unless indicated otherwise in a credit line to the material. If material is not included in the article’s Creative Commons license and your intended use is not permitted by statutory regulation or exceeds the permitted use, you will need to obtain permission directly from the copyright holder.
To view a copy of this license, visit https://creativecommons.org/licenses/by/4.0/

Manuscript NCOMMS-25-104081-T

Astroglial disinhibition of cortical circuits disrupts cognition via kynurenic acid

Viktor Beilmann, Johanna Furrer, Sina M. Schalbetter, Ron Schaer, Edoardo Tiziani, Kim D. Ferrari, Felisa Herrero, Celine Heeb, Alexandra von Faber-Castell, Jacqueline Condrau, Ulrike Weber-Stadlbauer, Matthias T. Wyss, Aiman S. Saab, Sarah Beggiato, Urs Meyer, Bruno Weber, Tina Notter

Responses to Reviewers' Comments

REVIEWER 1

1. The revised manuscript shows a substantial improvement over the previous version, both in overall clarity and in the strength of several newly added datasets. In particular, the expanded cellular analyses and the cell-type–targeted manipulation supporting an astrocytic source of KYNA significantly reinforce the main narrative. I congratulate the authors for the effort and for seriously engaging with many of the prior concerns. However, a few key issues remain only partially addressed, mainly regarding mechanistic specificity, statistical rigor for sex-related claims, and interpretability of variability/expression–phenotype relationships. These points still limit the strength of some causal statements and should be addressed.

Response: We sincerely thank the reviewer for their thoughtful evaluation of our manuscript and for the very encouraging feedback. We greatly appreciate the recognition that the clarity of the manuscript has improved and that the newly added datasets have strengthened the overall narrative. We are grateful for the reviewer's acknowledgment of our efforts to address the prior concerns. We also appreciate the reviewer's continued constructive critique. The points raised are well taken, and we have addressed each of them in detail below, with additional analyses, clarifications, and revisions to strengthen the causal interpretations where appropriate.

2. The rationale for using Gq-DREADDs is reasonable, but the manuscript still does not test whether the reported behavioral/circuit effects are specific to PLC–IP3/Ca^{2+} signaling versus other astrocytic signaling pathways (e.g., Gi/Gs/cAMP). The added MIA model supports broader relevance but does not resolve this mechanistic specificity question. At minimum, mechanistic language should be tempered.

Response: We thank the reviewer for this important comment and agree that directly testing whether the observed effects are specifically mediated by astrocytic PLC-IP3/Ca^{2+} signaling, rather than by other astrocytic pathways, would provide valuable mechanistic clarification. However, such pathway-specific dissection would require additional animal experiments and corresponding ethical approval, which would go beyond the scope of the present study and substantially delay publication.

As the primary objective of this study was to establish the astrocytic contribution to KYNA-dependent circuit and behavioral effects, we believe that the current dataset is sufficiently novel and rigorous, even without a more detailed dissection of the specific intracellular pathways involved. We consider the present findings to provide a strong foundation for future studies aimed at systematically distinguishing between PLC-IP3/Ca^{2+} signaling and other astrocytic signaling mechanisms (e.g., Gi/Gs-cAMP pathways).

Accordingly, we have added additional sentences to the Discussion to explicitly acknowledge this limitation and to outline it as an important direction for future research. In

addition, we have tempered the mechanistic language throughout the manuscript to avoid overattribution to PLC-IP3/Ca^{2+} signaling where alternative astrocytic pathways may also contribute. Accordingly, we acknowledge that direct evidence for PLC-IP3/Ca^{2+} specificity is not provided here.

3. Claims that effects are “similar” across sexes are not supported by formal statistics (e.g., sex × treatment interaction or direct male–female comparisons). Please either provide these tests.

Response: We thank the reviewer for raising this important point. In the original version of the manuscript, we analyzed the data presented in Figure 2 (males) and Supplementary Figure S6 (females) separately, as the majority of experiments were conducted in male animals. In response to the reviewer’s comment, we have now performed additional full-factorial ANOVAs including sex as an independent factor. The results of these analyses are summarized in Supplementary Table S1 and have been incorporated into the Results section. With the exception of PPI, there were no significant sex × treatment interactions in any other behavioral or cognitive test. In the PPI test, a significant pulse × treatment × sex interaction was observed, reflecting that females exhibited a significant deficit only under the 120-dB_A pulse condition, whereas males showed an overall significant reduction in PPI across conditions. Hence, even though females displayed a more restricted pattern of impairment, both sexes exhibited treatment-related deficits in PPI. Therefore, these additional analyses support our initial conclusion that the behavioral and cognitive effects induced by chemogenetic activation of prefrontal astrocytes are comparable between male and female animals.

4. The manuscript does not address whether phenotype magnitude relates to the extent of astrocyte DREADD expression/activation in the DREADD cohorts. The MIA GFAP correlation is interesting but not a direct substitute. A correlation between expression/coverage (or activation readouts) and behavioral/cellular outcomes would strengthen interpretation.

Response: We thank the reviewer for this important and insightful comment and agree that determining whether the degree of astrocyte activation correlates with the magnitude of behavioral and cellular outcomes would strengthen the mechanistic link between prefrontal astrocytes, circuit alterations, and cognitive deficits.

To address this point, we performed additional immunohistochemical analyses using tissue from cohort 12 (behaviorally tested hM3DGq-expressing male mice that were also used for the c-Fos analysis following pharmacological inhibition of KATII). Astrocyte activation was indexed by quantifying c-Fos expression in astrocytes (as shown by Erickson *et al.*, 2021, PMID: 32464636), together with changes in GFAP intensity. These astrocytic activation measures were then correlated with cognitive performance (temporal order memory) and neuronal activity (c-Fos expression in PV interneurons and CaMKII-positive neurons) following hM3DGq activation.

This new dataset is presented in Suppl. Fig. S9. The corresponding results are described in the revised Results section, and the Methods section has been updated accordingly. Importantly, these additional analyses revealed significant negative correlations between astrocyte activation measures and temporal order memory performance, as well as significant negative correlations between astrocyte activation and PV interneuron activity. In contrast, we identified a positive correlation between astrocyte activation and CaMKII-positive pyramidal neuron activity. Together, these additional data support the hypothesis that the magnitude of the behavioral and circuit phenotype is indeed directly related to the extent of astrocytic DREADD activation. These findings and their implications are now discussed in the revised manuscript.

REVIEWER 2

1. The manuscript "Astroglial disinhibition of cortical circuits disrupts cognition via kynurenic acid" by Beilmann et al. demonstrates that increased synthesis of KYNA in astrocytes can inhibit PV interneurons in the prefrontal cortex and cause cognitive impairments. The work is performed using targeted genetic manipulations and in vivo calcium recordings, thus providing relevant experimental evidence. Moreover, the authors include a mouse model of neurodevelopmental disorders and show that this mechanism is activated in this model. The authors have made significant additions compared with an earlier version of the paper and now provide a comprehensive study of astrocytic KYNA in the prefrontal cortex. The experiments are well performed and the manuscript is well written. The data are well presented and appropriate controls are used. The work is relevant in a complex field and provides an important piece of the puzzle of astrocyte–neuron interactions. I have a few minor comments:

Response: We thank the reviewer for the positive and encouraging evaluation of our manuscript. We greatly appreciate the recognition that the revised version provides a more comprehensive analysis of astrocytic KYNA in the prefrontal cortex and that the experiments, controls, and data presentation are clear and appropriate. We also sincerely appreciate the reviewer's view that our findings provide a meaningful contribution toward advancing the understanding of astrocyte-neuron interactions within this complex and evolving field.

2. The authors imply that increased KYNA is causative in cognitive dysfunction and part of the pathophysiological process. When KAT is reduced, the effect of DREADD-induced activation on cognition and c-Fos expression in PV neurons is abolished. However, the knockdown of KAT is partial and shows substantial variability, as does the exploration time in the cognitive test. If the authors still have available samples from the animals that underwent cognitive testing, the paper could be strengthened by showing a correlation between KYNA levels and cognitive deficits.

Response: We thank the reviewer for this thoughtful comment. We acknowledge that the KATII knockdown achieved in our experiments is partial and exhibits variability across animals. We also recognize variability in exploration time during cognitive testing. Importantly, however, the extent of variability in exploration time is comparable to that observed in other cohorts tested in the same behavioral paradigm (see Figures 2 and 5), suggesting that it is unlikely to be driven specifically by differences in KATII knockdown efficiency.

We agree that directly correlating KYNA levels with cognitive performance would strengthen the mechanistic link between KYNA and behavioral deficits. Unfortunately, we do not have remaining fresh tissue from the behaviorally tested animals that would allow reliable KYNA quantification and subsequent correlation analyses. Accordingly, we have tempered the relevant causal language in the manuscript.

3. In addition, in the MIA model, a correlation between KYNA, GFAP, and plasma KYN is shown (Fig. 5), but not in controls. If KYNA is causative, such a correlation should exist regardless of disease model, and the authors could consider merging control and MIA animals in the same graph.

Response: We thank the reviewer for this insightful comment. In line with the reviewer's suggestion, we have now performed additional partial correlations across the full dataset, controlling for prenatal treatment. These analyses reveal significant correlations between KYN, GFAP, and behavioral measures when considering all animals together (see Suppl. Fig. S11b-d). These results support the notion that KYN levels may generally associate with astrocyte activation and cognitive outcomes beyond the pathological context of the MIA.

4. The statistics for PPI are reported differently across experiments and are not very convincing. In female mice (Suppl. Fig. S6f), significance is observed at a specific stimulus

intensity, whereas in male mice significance is reported for the merged dataset (Fig. 2). This should be clarified, and the actual behavioral effect should be discussed.

Response: We thank the reviewer for raising this important point and agree that our initial description of the PPI analysis across sexes was incomplete. We have therefore amended this in the revised manuscript and now report all relevant statistical analyses.

Specifically, in females (see Suppl. Fig. S6), significant PPI deficits were observed only under conditions in which 120 dBA stimuli were used as pulses. This pulse-specific effect is supported by a main effect of treatment ($F_{(1,18)}$ = 7.82, p < 0.05) in a 2 × 3 (treatment × prepulse) repeated-measures ANOVA conducted specifically for the 120 dBA pulse condition, which was performed following the presence of a significant main effect of pulse ($F_{(2,36)}$ = 6.84, p < 0.01) and a treatment × pulse interaction ($F_{(2,36)}$ = 3.63, p < 0.05) in the preceding full-factorial 2 × 3 × 3 (treatment × pulse × prepulse) repeated-measures ANOVA.

In males (see figure legend of Fig. 2d), a significant overall reduction in %PPI was observed. Importantly, this effect was not based on a merged dataset (i.e., averaging across all prepulse and pulse stimuli), but instead reflects a significant main effect of treatment ($F_{(1,18)}$ = 9.98, p < 0.01) in the full-factorial 2 × 3 × 3 (treatment × prepulse × pulse) repeated-measures ANOVA.

5. The amounts of KYN and KYNA are reported using different units in Fig. 1 and Supplementary Fig. S7 so the results cannot be compared. In the first experiment, DREADD activation of astrocytes reduces KYN levels, whereas in the second experiment it does not. This discrepancy should be addressed.

Response: We thank the reviewer for noting this issue and apologize for the inconsistency in how these data were reported. In the dataset shown in Fig. S7, tissue weights could not be obtained, which resulted in different units being used (pmole/mg tissue in Fig. 2 and nM in injected solution in Fig. S7). In addition, direct comparison between the two datasets is complicated by methodological differences: in Fig. 2, KYN and KYNA were measured from pooled PFC tissue of two mice, whereas in Fig. S7 measurements were obtained from the PFC of single animals. In both cases, the tissue was dissolved in the same volume of methanol. To improve transparency, we now provide the concentrations (nM) measured in the injected solutions of both dataset in the Source Data. These methodological differences have now been explicitly clarified in the Methods section.

Regarding the second point, we believe there may have been a misunderstanding. Contrary to the reviewer's impression, our data did not reveal a significant reduction in KYN levels following astrocyte activation. Although a non-significant trend toward lower KYN levels is visible in the first experiment, we have intentionally avoided discussing non-significant trends in the main manuscript. We currently do not have a clear explanation for why a similar trend is not apparent in the second dataset. Potential contributing factors include the different sampling strategy (pooled vs. single PFC), differences in normalization due to unavailable tissue weights, and increased biological and technical variability in the single-sample measurements. Given the absence of statistically significant changes, we have avoided speculative interpretation in the manuscript. We hope the reviewer agrees that refraining from discussing non-significant trends is the most appropriate and rigorous approach.

6. There is a vast literature on the kynurenine pathway, and the roles of individual metabolites are complex and difficult to disentangle. Moreover, the authors mention that KYNA inhibits NMDA receptors, but several other receptors may also be involved. This should be more clearly acknowledged in introduction and discussion.

Response: We thank the reviewer for this important point and agree that our original description of KYNA targets was overly narrow. We recognize that we did not sufficiently acknowledge the complexity of the kynurenine pathway and the range of receptors that may be influenced by KYNA. In the revised manuscript, we have explicitly adjusted the text to acknowledge additional receptor targets and to better reflect the broader literature on the diverse actions of kynurenine pathway metabolites.

REVIEWER 3

1. The authors have addressed my main comments. Although the manuscript has improved significantly, it would be important to discuss the key role of astrocytes metabolism on cognitive function. This will increase the translational impact of the study.

Response: We thank the reviewer for this constructive suggestion and fully agree that discussing the key role of astrocyte metabolism in cognitive function strengthens the translational impact of our study. In the revised manuscript, we have therefore added the following paragraph to the Discussion part:

> *Alongside our findings, there is a large body of literature demonstrating the importance of astrocytic metabolism and astrocytic-neuronal metabolic coupling in shaping brain activity and cognitive functions (Bonvento and Bolaños 2021; Bolaños and Magistretti 2025; Barros et al., 2025). For example, sustained astrocytic mitochondrial ROS production modulates brain metabolism and supports cognitive performance (Vicente-Gutierrez et al., 2019, Morant-Ferrando et al., 2023). KYNA has also been reported to exert antioxidant effects through free radical scavenging, thereby mitigating oxidative stress (Lugo-Huitrón et al., 2011). Extending this framework, we identify astrocyte-derived KYNA in the PFC as a metabolic factor mediating cognitive dysfunction. Future work will determine whether KYNA primarily disrupts cognition via neurotransmitter receptor antagonism and subsequent cortical disinhibition, or whether it additionally intersects with astrocytic ROS-dependent pathways to shape neural circuit excitability.*
